# Impact of atrial fibrillation on clinical outcomes in 28,492 patients following open-heart cardiac valve surgery: A statewide population-linkage study

**DOI:** 10.1186/s12916-026-04953-2

**Published:** 2026-05-27

**Authors:** Jia Yi Anna Ne, Clara K Chow, Vincent Chow, Karice Hyun, Leonard Kritharides, David Brieger, Austin Chin Chwan Ng

**Affiliations:** 1 Department of Cardiology, Concord Repatriation General Hospital, Concord, NSW Australia; 2 Department of Cardiology, Westmead Hospital, Westmead, NSW Australia; 3 Westmead Applied Research Centre, The University of Sydney, Westmead, NSW Australia; 4 The University of Sydney, Faculty of Medicine and Health, Camperdown, NSW Australia; 5 ANZAC Research Institute, The University of Sydney, Concord, NSW, Australia

**Keywords:** Atrial fibrillation, Clinical outcomes, Aortic valve surgery, Mitral valve surgery

## Abstract

**Background:**

Atrial fibrillation (AF) is common after cardiac surgery. Few studies compared clinical outcomes associated with AF between patients undergoing aortic valve surgery (AVSx) versus mitral valve surgery (MVSx).

**Methods:**

Patients who had open-heart cardiac valve surgery (1- July-2003 to 31-March-2021) identified from the Admitted-Patient-Data-Collection database in Australia were stratified by AF status (No-AF vs. New-AF vs. Prior-AF during index valve surgery) and followed up to 31-March-2022. Multivariable Cox regression and Fine-Gray competing risk analyses were performed to assess the association of AF status on all-cause mortality and non-fatal outcomes respectively.

**Results:**

The cohort comprised 28,492 patients (whole cohort median age 71.6yrs [interquartile range 62.7–78.3yrs]; 65.6% males): AVSx, *n* = 18,949, median age 73.3 [IR 65.4–79.4yrs], 67.8% males; MVSx, *n* = 9543, MVSx: median age 67.6 [IR 58.2–75.5yrs], 61.2% males. During a median 6.58yrs (3.4–10.5yrs) follow-up, Prior-AF and New-AF patients had significantly higher all-cause mortality (AVSx-Prior-AF: 57.9% vs. AVSx-New-AF: 41.5% vs. AVSx-No-AF: 32.8%; MVSx-Prior-AF: 41.0% vs. MVSx-New-AF: 29.8% vs. MVSx-No-AF: 22.4%) (both logrank *P* < 0.001). In the AVSx subgroup, both New-AF and Prior-AF were independently associated with all-cause mortality (aHR = 1.16, 95%CI = 1.10–1.22; aHR = 1.69, 95%CI = 1.59–1.79 respectively) compared to No-AF patients. In the MVSx subgroup, only Prior-AF was associated with increased all-cause mortality (aHR = 1.47, 95% CI = 1.33–1.61), all *P* < 0.001. Ischaemic stroke was significantly higher in the AVSx New-AF and AVSx-Prior-AF subgroups.

**Conclusions:**

Patients undergoing valve surgery have different risks of adverse clinical outcomes, with target valve and baseline AF status being associated with these outcomes.

**Supplementary Information:**

The online version contains supplementary material available at https://doi.org/10.1186/s12916-026-04953-2.

## Background

Atrial fibrillation (AF) is the most common complication after cardiac surgery, occurring in 30% of patients who had cardiac surgery [1]. Although the incidence of postoperative AF (POAF) varies considerably between studies [2], it is generally higher in patients who undergo cardiac valve surgery compared to those who undergo coronary artery bypass graft (CABG) surgery, occurring in 20–40% in patients undergoing CABG and in 40–50% in patients undergoing cardiac valve surgery [3, 4].

Despite POAF being often viewed as a relatively benign event triggered by the stress and subsequent catecholamine release from surgery [5], there is emerging evidence suggesting POAF can have adverse sequelae especially in elderly patients and those with left ventricular dysfunction [6]. POAF has been associated with a higher mortality risk and other adverse clinical outcomes including congestive heart failure, stroke, myocardial infarction, recurrent AF, renal dysfunction and haemodynamic instability, leading to prolonged hospitalisation and higher healthcare costs [7, 8].

Meta-analyses showing increased mortality and morbidity risk in patients with POAF following cardiac valve surgery were heterogenous with regards to the included studies, with some including patients undergoing multiple valve surgeries and valve surgery with concomitant CABG [9, 10], or including relatively small cohort sizes for the individual studies [9]. In addition, the lack of consensus on defining POAF by professional societies [11, 12] has given rise to differing definitions of POAF and differing exclusion criteria. There is also a paucity of studies defining the impact of new-onset versus prior-AF on fatal and non-fatal clinical outcomes during long-term follow-up and few studies have compared the prevalence and impact of POAF on clinical outcomes between patients who underwent open-heart aortic valve surgery (AVSx) versus mitral valve surgery (MVSx) [13, 14]. Bridging these gaps in the literature will have important implications regarding the clinical relevance of POAF and potentially inform future treatment paradigms for different surgical groups.

The present study aims to compare the rates of new-onset versus prior-AF, clinical characteristics and outcomes in patients who underwent open-heart AVSx and MVSx identified from a large statewide population cohort database and examine the impact of AF status on patients’ outcomes.

## Methods

### Study population

The Centre for Health Record Linkage (CHeReL) holds one of the largest data linkage systems in Australia containing linked health data of users of the health system in the state of New South Wales (NSW) [15], Australia’s largest population state – approximately 8.153 million residents, 30% of Australian population [16]. From the Admission Patient Data Collection (APDC) administrative database, one of CHeReL-derived key databases which includes ≥ 97% of all healthcare facilities in the state, we identified consecutive admissions for cardiac valve surgery between 1-July-2003 and 31-March-2021 [15]. For this study, we focussed on adult patients (≥ 18yo) who underwent open-heart AVSx or MVSx. Patients were excluded if they had known pre-existing prosthetic valve, or if they had undergone concomitant surgery on tricuspid, pulmonary, mitral valve (in the case of AVSx group) or aortic valve (in the case of MVSx group) during the index open-heart AVSx or MVSx admission; concomitant CABG was allowed and adjusted for in our analysis (see Supplementary Figure S1 for study cohort derivation). The study cohort was limited to only NSW residents to minimise incomplete tracking of outcomes. An important aspect of our study was to compare patient’s outcomes based on different AF categories (New-AF and Prior-AF) versus No-AF status at the time of their index valve surgery admission (aortic vs. mitral valve surgery). A linkage look-back within the APDC database to year-2001 was performed to identify prior history of AF and follow-up was until 31-March-2022. For the purposes of this study, the definition of AF includes atrial flutter and encompasses the following codes I48, I48.0, I48.1, I48.2, I48.3, I48.4, I48.9 listed under the International Statistical Classification of Diseases and Related Health Problems, Tenth Revision, Australian Modification (ICD-10AM) system. The AF status of patients at the time of their index open-heart valve surgery are defined as follows: No-AF refers to patients without AF; New-AF refers to patients with new-onset AF during index cardiac valve surgery admission and no prior history of AF; and Prior-AF refers to patients with a history of AF.

### Data sources

Data variables obtained from the APDC database for each hospital admission include age, sex, region of residence defined by the recorded Statistical Area Level 2 code [17] for the patient, time/date of admission, admission referral source, length of admission, and whether the patient died in hospital. The Australian Statistical Geography Standard – Remoteness Area (ASGS-RA) is a set of geographical data used by the Australian Bureau of Statistics and has five levels of remoteness as a measure of relative access to services [17]. For this study, we re-defined major cities as metropolitan group (reference cohort), followed by inner-regional group, and lastly outer-regional/remote group. In addition, we derived the index of relative socio-economic advantage and disadvantage (IRSAD) score [18] for each patient based on their region of residence. This is a summation score reflecting the socioeconomic conditions of people within an area and includes both relative advantage and disadvantage measures. A higher score reflects a relative lack of disadvantage and greater advantage in general.

The primary and all secondary diagnoses (potentially up to 50 secondary diagnoses) associated with each admission were retrieved from the APDC database. Each diagnosis was coded in the APDC database according to the ICD-10AM system. Comorbidities of interest extracted for this study and rates of concomitant procedures of interest performed during admission including CABG, percutaneous coronary intervention (PCI), implantation of either a permanent pacemaker (PPM) or cardioverter defibrillation (ICD) are shown in Table 1 (see Supplementary Table S1 for relevant ICD-10AM codes and the Australian Classification of Health Interventions procedural codes). In addition, the overall comorbidity burden of each patient was semi-quantified using the Charlson comorbidity index (CCI) [19], which is a summation score based on 17 medical conditions with varying assigned weights (non-age-adjusted). A score of 1 was given to each of the following conditions: myocardial infarction, congestive heart failure, peripheral vascular disease, stroke, dementia, chronic pulmonary disease, connective tissue disease, peptic ulcer disease, mild liver disease, and diabetes (without organ damage); 2 for hemiplegia, moderate-severe renal disease, any tumour (within last 5 years), lymphoma, leukaemia, or diabetes with organ damage; 3 for moderate-severe liver disease; and a score of 6 for metastatic solid tumour and acquired immune deficiency syndrome. The CHA_2_DS_2_VASc score was used to assess the stroke risk of patients and was calculated by adding points for each of the following conditions based on ICD-10-AM codes: heart failure: 1-point; hypertension: 1-point; age ≥ 75 year old: 2 points; Age 65–74 year old: 1-point; diabetes: 1-point; stroke / transient ischaemic attack / thromboembolism: 2-points; vascular disease (ischaemic heart disease including prior myocardial infarction, peripheral vascular disease): 1-point; sex category (female): 1-point.

**Table 1:** a. Baseline characteristics of open-heart aortic valve surgery cohort stratified by atrial fibrillation status during admission*. b. Baseline characteristics of open-heart mitral valve surgery cohort and atrial fibrillation status during admission*

a			
Parameters	Aortic valve surgery (N=18949)		
	No-AF group (N=9017, 47.6%)	New-AF group (N=6874, 36.3%)	Prior-AF group(N=3058, 16.1%)
Age, years	70.6 (61.2–77.6)	74.5 (68.1–80.1) †	76.8 (70.6–81.9) †‡
Males	6118 (67.8%)	4627 (67.3%)	2100 (68.7%)
Length of stay, days	9.0 (8.0–13.0)	12.0 (9.0–17.0) †	13.0 (9.0–20.0) †‡
Referred from ED	726 (8.1%)	540 (7.9%)	267 (8.7%)
ICU admission	8065 (89.4%)	6165 (89.7%)	2762 (90.3%)
IRSAD score	992.6 (936.3–1057.7)	990.5 (935.1–1 055.5)	989.8 (933.2–1 063.1)
ASGSRA status			
Metropolitan	6401 (71.0%)	4656 (67.7%) †	2115 (69.2%) †
Regional	2136 (23.7%)	1763 (25.6%) †	738 (24.1%) †
Remote	474 (5.3%)	453 (6.6%) †	205 (6.7%) †
**Cardiac risk factors**			
Hypertension	3813 (42.3%)	3208 (46.7%) †	1429 (46.7%) †
Hyperlipidaemia	982 (10.9%)	733 (10.7%)	295 (9.6%)
Diabetes	2131 (23.6%)	1665 (24.2%)	847 (27.7%) †‡
Current/ex-smoker	4173 (46.3%)	3151 (45.8%)	1325 (43.3%) †‡
**Co-morbidities**			
CHF	816 (9.0%)	913 (13.3%) †	628 (20.5%) †‡
Venous thromboembolism	69 (0.8%)	96 (1.4%) †	45 (1.5%) †
-DVT	52 (0.6%)	83 (1.2%) †	40 (1.3%) †
-PE	21 (0.2%)	18 (0.3%)	8 (0.3%)
Ischaemic heart disease	3554 (39.4%)	3166 (46.1%) †	1406 (46.0%) †
Prior CABG or PCI	655 (7.3%)	460 (6.7%)	312 (10.2%) †‡
Ischaemic stroke	122 (1.4%)	138 (2.0%) †	83 (2.7%) †‡
Haemorrhagic stroke	14 (0.2%)	17 (0.2%)	7 (0.2%)
Peripheral vascular disease	753 (8.4%)	624 (9.1%)	318 (10.4%) †‡
Malignancy	59 (0.7%)	47 (0.7%)	18 (0.6%)
Sleep apnoea	184 (2.0%)	125 (1.8%)	79 (2.6%) ‡
Chronic kidney disease	496 (5.5%)	579 (8.4%) †	371 (12.1%) †‡
Chronic pulmonary disease	407 (4.5%)	395 (5.7%) †	237 (7.8%) †‡
Neurodegenerative disease	46 (0.5%)	41 (0.6%)	27 (0.9%)
Major bleed	607 (6.7%)	633 (9.2%) †	258 (8.4%) †
Charlson comorbidity index	0.95 ± 1.51	1.14 ± 1.65 †	1.43 ± 1.82 †‡
CHA_2_DS_2_VASc score	2.60 ± 1.55	3.09 ± 1.51 †	3.36 ± 1.47 †‡
**Concomitant procedures performed during index admission of open-heart cardiac valve surgery**			
CABG	3396 (37.7%)	2990 (43.5%) †	1218 (39.8%) †‡
PCI	24 (0.3%)	37 (0.5%) †	8 (0.3%)
PPM	355 (3.9%)	324 (4.7%) †	188 (6.1%) †‡
ICD	29 (0.3%)	33 (0.5%)	15 (0.5%)

b			
Parameters	Mitral valve surgery (N=9543)		
	No-AF group(N=3330, 34.9%)	New-AF group(N=3466, 36.3%)	Prior-AF group(N=2747, 28.8%)
Age, years	61.8 (51.4–71.0)	69.0 (60.7–76.2) †	71.3 (63.6–77.7) †‡
Males	2184 (65.6%)	2122 (61.2%) †	1534 (55.8%) †‡
Length of stay, days	9.0 (8.0–14.0)	12.0 (9.0–18.0) †	12.0 (9.0–18.0) †
Referred from ED	374 (11.2%)	386 (11.1%)	175 (6.4%) †‡
ICU admission	2950 (88.6%)	3062 (88.3%)	2404 (87.5%)
IRSAD score	1003.3 (941.8–1073.9)	1006.5 (942.8– 1073.9)	992.6 (936.2–1065.3) †‡
ASGSRA status			
Metropolitan	2512 (75.4%)	2560 (73.9%)	1941 (70.7%) †‡
Regional	650 (19.5%)	705 (20.3%)	632 (23.0%) †‡
Remote	166 (5.0%)	197 (5.7%)	174 (6.3%) †‡
**Cardiac risk factors**			
Hypertension	962 (28.9%)	1129 (32.6%) †	889 (32.4%) †
Hyperlipidaemia	268 (8.0%)	243 (7.0%)	179 (6.5%) †
Diabetes	379 (11.4%)	421 (12.1%)	421 (15.3%) †‡
Current/ex-smoker	1311 (39.4%)	1352 (39.0%)	1 057 (38.5%)
**Co-morbidities**			
CHF	454 (13.6%)	617 (17.8%) †	642 (23.4%) †‡
Venous thromboembolism	41 (1.2%)	56 (1.6%)	38 (1.4%)
-DVT	33 (1.0%)	49 (1.4%)	28 (1.0%)
-PE	13 (0.4%)	10 (0.3%)	12 (0.4%)
Ischaemic heart disease	936 (28.1%)	1153 (33.3%) †	881 (32.1%) †
Prior CABG or PCI	130 (3.9%)	112 (3.2%)	147 (5.4%) †‡
Ischaemic stroke	76 (2.3%)	91 (2.6%)	61 (2.2%)
Haemorrhagic stroke	16 (0.5%)	8 (0.2%) †	7 (0.3%)
Peripheral vascular disease	159 (4.8%)	160 (4.6%)	139 (5.1%)
Malignancy	15 (0.5%)	16 (0.5%)	23 (0.8%)
Sleep apnoea	45 (1.4%)	40 (1.2%)	51 (1.9%) ‡
Chronic kidney disease	135 (4.1%)	200 (5.8%) †	204 (7.4%) †‡
Chronic pulmonary disease	109 (3.3%)	164 (4.7%) †	159 (5.8%) †
Neurodegenerative disease	4 (0.1%)	11 (0.3%)	12 (0.4%) †
Major bleed	207 (6.2%)	315 (9.1%) †	206 (7.5%) †‡
Charlson comorbidity index	0.68 ± 1.35	0.78 ± 1.44 †	0.96 ± 1.52 †‡
CHA_2_DS_2_VASc score	1.89 ± 1.55	2.45 ± 1.62 †	2.71 ± 1.54 †‡
**Concomitant procedures performed during index admission of open-heart cardiac valve surgery**			
CABG	902 (27.1%)	1111 (32.1%) †	789 (28.7%) ‡
PCI	11 (0.3%)	29 (0.8%) †	8 (0.3%) ‡
PPM	91 (2.7%)	163 (4.7%) †	148 (5.4%) †
ICD	36 (1.1%)	47 (1.4%)	33 (1.2%)

Continuous variables are expressed as medians with interquartile range in brackets or mean ± SD; all others represent numbers of patients with values in brackets representing percentages.

AF, atrial fibrillation; ED, Emergency Department; ICU, intensive care unit; IRSAD score, Index of Relative Socioeconomic Advantage and Disadvantage score; ASGSRA, Australian Statistical Geography Standard Remoteness Area; CHF, congestive heart failure; DVT, deep vein thrombosis; PE, pulmonary embolism; CABG, coronary artery bypass graft; PCI, percutaneous coronary intervention; PPM, permanent pacemaker; ICD, implantable cardioverter defibrillator.

^*^No-AF represents patients without AF; New-AF represents patients with new AF during index admission for cardiac valve surgery and no prior history of AF; Prior-AF represents patients with a history of AF.

^†^P<0.05 compared to No-AF group

^‡^P<0.05 compared to New-AF group

### Clinical outcome endpoints

The all-cause mortality and morbidity outcomes of our cohort were tracked using linkage methods from the statewide death registry and the APDC database respectively until 31-March-2022. The ICD-10AM codes used to track the morbidity outcomes are defined in Supplementary Table S1. Our co-primary outcomes were major adverse cardiovascular events (MACE) and all-cause mortality post-admission. MACE was defined as all-cause mortality, admission for myocardial infarction, ischaemic stroke, congestive heart failure or coronary revascularisation (PCI or CABG). These outcomes were selected to facilitate comparison with other studies [20]. Our exploratory secondary endpoints include rates of non-fatal cardiovascular outcomes that were clinically significant requiring rehospitalisation: AF (primary / secondary diagnosis), acute myocardial infarction, congestive heart failure, ischaemic stroke, haemorrhagic stroke, venous thromboembolism, peripheral arterial thromboembolism, bleeding, and non-fatal MACE (defined as admission for myocardial infarction, ischaemic stroke, heart failure or coronary revascularisation).

The study protocol conforms to the ethical guidelines of the 2013 Declaration of Helsinki. Ethics approval was granted by the NSW Population and Health Services Research Ethics Committee (PHSREC), reference number: 2019/ETH01790. The Ethics Committees granted a waiver of the usual requirement for the consent of the individual to the use of their health information. All patient data were de-identified and analysed anonymously.

### Statistical analysis

All continuous variables were expressed as mean ± standard deviation (SD) or median (interquartile range [IQR]). Categorical variables were presented in absolute numbers and proportions. Comparison between groups were performed using Mann-Whitney-U test (2 groups) or Kruskal-Wallis test (3 groups) for non-parametric continuous variables respectively, and chi-square tests for dichotomous variables. Kaplan-Meier methods were used to determine survival rates. The variables extracted from the APDC database and shown in Table 1 were chosen based on prior literature and their clinical plausibility pertaining to outcomes for this study cohort. To determine the association of baseline AF status of these patients undergoing open-heart valve surgery on the subsequent primary clinical outcomes of MACE and all-cause mortality during follow-up, Cox proportional hazards regression method was used to adjust for differences in patients’ baseline characteristic parameters. In addition, the strength of the association of the baseline variables with our primary clinical outcome analyses were presented. To ensure robustness of the multivariable Cox regression analysis, only univariables with *P* < 0.05 were included. Multivariable Cox proportional hazards regression was performed to assess the association of AF status on MACE and all-cause mortality during follow-up. Variables from univariable analyses with *P* < 0.05 were included in the multivariable Cox regression analysis. The proportional hazards assumption was checked with log-minus-log plots. To avoid potential multicollinearity, a tolerance of > 0.4 (equating to a Variance Inflation Factor of > 2.5) was set. In addition, sensitivity analysis on the primary outcomes of MACE and all-cause mortality was performed by removing patients who underwent concomitant CABG. In order to determine if the relationship between AF status and MACE or all-cause mortality differed by type of cardiac valve surgery, we also performed interaction analyses between AF status and type of cardiac valve surgery on the primary outcomes in our multivariable analyses. To account for the competing risk of death in assessing the impact of AF status on non-fatal cardiovascular endpoints, Fine-Gray competing risk analysis was performed. We also performed additional exploratory competing risk regression analyses to determine the impact of AF status on the risk of ischaemic stroke in both AVSx and MVSx subgroups with elevated CHA_2_DS_2_VASc score (defined as ≥ 1).

All analyses were performed using SPSS v29 (IBM, USA). A two-tailed probability value < 0.05 was considered statistically significant.

## Results

### Baseline characteristics

The study cohort comprised 28,492 patients (median age 71.6yrs [IQR: 62.7–78.3yrs; 65.6% males): AVSx, *n* = 18949; MVSx, *n* = 9543 (Table 1a and 1b respectively). The rates of New-AF in the AVSx and MVSx subgroups during the index surgery admission were similar (36.3%). In the AVSx subgroup, 16.1% had Prior-AF whereas 28.8% had Prior-AF in the MVSx subgroup (*P* < 0.001). Within each AVSx and MVSx subgroups (Table 1a and 1b respectively), patients with New-AF and Prior-AF were significantly older and had a longer length of stay compared to patients with No-AF (*P* < 0.001). In addition, within each AVSx and MVSx subgroup, patients with Prior-AF were older, had a higher mean CCI score and CHA_2_DS_2_VASc score as well as a higher prevalence of diabetes, congestive heart failure and chronic kidney disease compared to patients with New-AF.

When comparing patients who underwent AVSx to those who had MVSx (Supplementary Table S2), the former group who had New and Prior-AF were significantly older and had higher concomitant CABG rates compared to patients who underwent MVSx (*P* < 0.001). In addition, compared to patients who underwent MVSx, patients who underwent AVSx had a higher frequency of cardiac risk factors such as hypertension, hyperlipidaemia and diabetes, stratified by AF status. While MVSx patients had a higher prevalence of comorbidities such as congestive heart failure compared to AVSx patients, AVSx patients had a higher prevalence of ischaemic heart disease and peripheral vascular disease compared to MVSx patients respectively. The frequency of non-cardiac comorbidities such as chronic kidney disease, obstructive sleep apnoea and chronic pulmonary disease were higher in the AVSx patients compared to MVSx patients. AVSx patients also had a higher mean CCI score and CHA_2_DS_2_VASc score compared to MVSx patients, reflecting an overall higher comorbidity burden (Supplementary Table S2).

### Crude rates of clinical endpoints

**Fig. 1:**
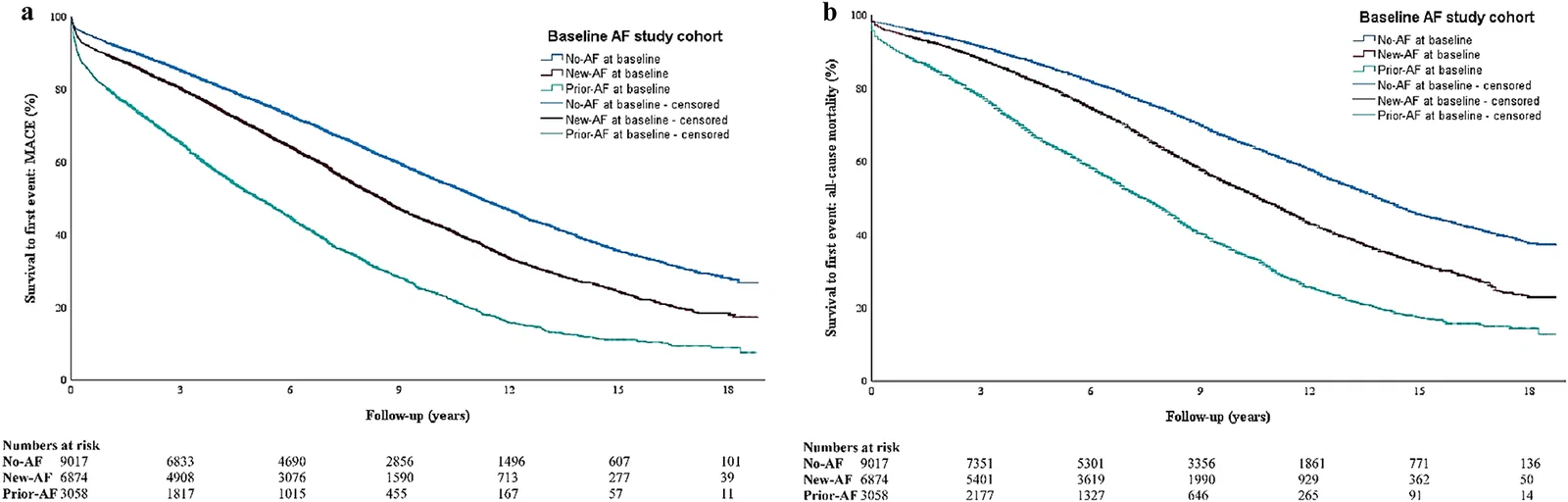
(**a**) Kaplan-Meier survival curves for MACE in aortic valve surgery subgroup stratified by AF status*. **b** Kaplan-Meier survival curves for all-cause mortality in aortic valve surgery subgroup stratified by AF status*. Figure 1 shows the Kaplan-Meier curves of the aortic valve surgery study cohort stratified by baseline AF status.The blue line represents No-AF at baseline, the red line represents New-AF at baseline and the green line represents Prior-AF at baseline groups. The survival curves differed significantly (logrank P < 0.001).AF, atrial fibrillation; MACE, major adverse cardiovascular events defined as all-cause mortality or rehospitalisation with a primary diagnosis of acute myocardial infarction or ischaemic cerebrovascular accident or heart failure or revascularisation (percutaneous coronary intervention or coronary artery bypass graft). *The study cohort was stratified by baseline AF status: No-AF group represents patients without prior history of AF or new AF documented during index aortic valve surgery admission; New-AF group represents patients with new AF documented during index aortic valve surgery admission and no prior history of AF; Prior-AF group represents patients with a documented history of AF prior to the index aortic valve surgery admission.

**Fig. 2:**
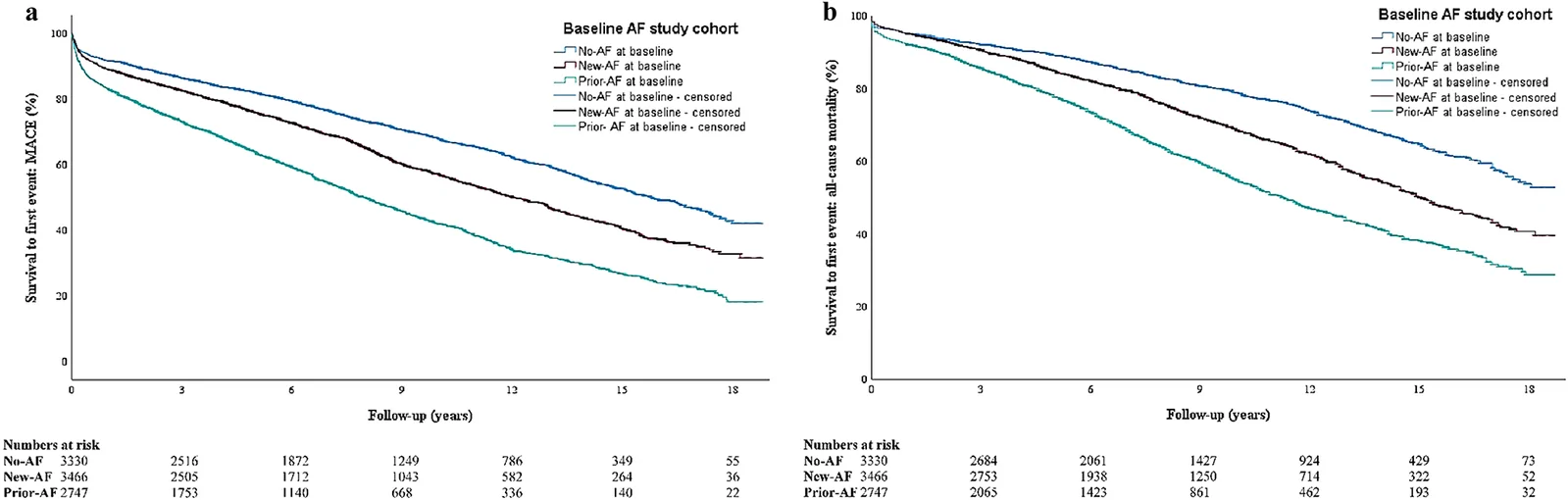
(**a**) Kaplan-Meier survival curves for MACE in mitral valve surgery subgroup stratified by AF status*. **b** Kaplan-Meier survival curves for all-cause mortality in mitral valve surgery subgroup stratified by AF status*. Figure 2 shows the Kaplan-Meier curves of the mitral valve surgery study cohort stratified by baseline AF status.The blue line represents No-AF at baseline, the red line represents New-AF at baseline and the green line represents Prior-AF at baseline groups.The survival curves differed significantly (logrank P < 0.001).AF, atrial fibrillation; MACE, major adverse cardiovascular events defined as all-cause mortality or rehospitalisation with a primary diagnosis of acute myocardial infarction or ischaemic cerebrovascular accident or heart failure or revascularisation (percutaneous coronary intervention or coronary artery bypass graft).*The study cohort was stratified by baseline AF status: No-AF group represents patients without prior history of AF or new AF documented during index mitral valve surgery admission; New-AF group represents patients with new AF documented during index mitral valve surgery admission and no prior history of AF; Prior-AF group represents patients with a documented history of AF prior to the index aortic valve surgery admission.

Clinical outcomes of the cohort are shown in Table 2. During a median follow-up of 6.58yrs (3.42–10.5yrs) for all-cause mortality and 5.66yrs (2.58–9.48 yrs) for MACE, Prior-AF and New-AF patients had significantly higher all-cause mortality and MACE compared to No-AF patients (both logrank *P* < 0.001). Non-fatal outcomes such as non-fatal MACE, atrial fibrillation, acute myocardial infarction, congestive heart failure, stroke, venous thromboembolism, peripheral arterial thromboembolism and bleeding differed significantly both between as well as within the AVSx and MVSx subgroups (*P* < 0.001). The Kaplan-Meier curves showing survival to first event MACE and all-cause mortality for the AVSx and MVSx subgroups stratified by AF status are shown in Figs. 1 and 2 respectively.

**Table 2:** Clinical outcomes of study cohort stratified by type of open-heart cardiac valve surgery and atrial fibrillation status during admission

Post-discharge clinical outcome endpoints *	Aortic valve surgery (*N* = 18949)			Mitral valve surgery (*N* = 9543)		
	No-AF group (*N* = 9017, 47.6%)	New-AF group (*N* = 6874, 36.3%)	Prior-AF group (*N* = 3058, 16.1%)	No-AF group (*N* = 3330, 34.9%)	New-AF group (*N* = 3466, 36.3%)	Prior-AF group (*N* = 2747, 28.8%)
Atrial fibrillation (Primary diagnosis)	610 (6.8%; 0.91)	1034 (15.0%; 2.46) ¶	685 (22.4%; 4.77) ¶#	385 (11.6%; 1.51) †	746 (21.5%; 3.42) ‡¶	767 (27.9%; 5.32) §¶#
Atrial fibrillation (Primary/secondary diagnosis)	1622 (18.0%; 2.57)	2781 (40.5%; 8.09) ¶	1821 (59.5%; 19.06) ¶#	734 (22.0%; 3.10) †	1473 (42.5%; 8.20) ‡¶	1657 (60.3%; 17.04) §¶#
Acute myocardial infarction	461 (5.1%; 0.67)	333 (4.8%; 0.71) ¶	189 (6.2%; 1.08) ¶#	137 (4.1%; 0.50) †	137 (4.0%; 0.53) ‡¶	119 (4.3%; 0.63) §¶#
Congestive heart failure	1034 (11.5%; 1.54)	1094 (15.9%; 2.48) ¶	833 (27.2%; 5.31) ¶#	321 (9.6%; 1.20) †	513 (14.8%; 2.08) ‡¶	647 (23.6%; 3.82) §¶#
Stroke						
Ischaemic	459 (5.1%; 0.67)	417 (6.1%; 0.90) ¶	226 (7.4%; 1.30) ¶#	181 (5.4%; 0.66) †	185 (5.3%; 0.71) ‡¶	181 (6.6%; 0.97) §¶#
Haemorrhagic	163 (1.8%; 0.23)	138 (2.0%; 0.29) ¶	65 (2.1%; 0.36) ¶#	55 (1.7%; 0.20) †	72 (2.1%; 0.27) ‡¶	66 (2.4%; 0.34) §¶#
Venous thromboembolism	173 (1.9%; 0.25)	119 (1.7%; 0.25) ¶	50 (1.6%; 0.28) ¶#	63 (1.9%; 0.23) †	62 (1.8%; 0.24) ‡¶	38 (1.4%; 0.20) §¶#
Pulmonary embolism	124 (1.4%; 0.18)	88 (1.3%; 0.19) ¶	33 (1.1%; 0.18) ¶#	50 (1.5%; 0.18) †	47 (1.4%; 0.18) ‡¶	27 (1.0%; 0.14) §¶#
Deep vein thrombosis	50 (0.6%; 0.07)	33 (0.5%; 0.07) ¶	19 (0.6%; 0.11) ¶#	14 (0.4%; 0.05) †	16 (0.5%; 0.06) ‡¶	11 (0.4%; 0.06) §¶#
Peripheral arterial thromboembolism	30 (0.3%; 0.04)	14 (0.2%; 0.03) ¶	10 (0.3%; 0.55) ¶#	4 (0.1%; 0.01) †	12 (0.3%; 0.05) ‡¶	15 (0.5%; 0.08) §¶#
Total bleeding events	2449 (27.2%; 4.08)	2039 (29.7%; 5.09) ¶	1099 (35.9%; 7.68) ¶#	794 (23.8%; 3.30) †	941 (27.1%; 4.20) ‡¶	843 (30.7%; 5.28) §¶#
In-hospital mortality	139 (1.5%)	169 (2.5%) ¶	155 (5.1%) ¶#	95 (2.9%) †	69 (2.0%) ¶	98 (3.6%) §#
All-cause mortality	2961 (32.8%; 4.28)	2854 (41.5%; 6.11) ¶	1771 (57.9%; 10.03) ¶#	747 (22.4%; 2.71) †	1034 (29.8%; 3.97) ‡¶	1126 (41.0%; 5.94) §¶#
MACE	3808 (42.2%; 6.08)	3465 (50.4%; 8.39) ¶	2103 (68.8%; 14.58) ¶#	1063 (31.9%; 4.22) †	1372 (39.6%; 5.89) ‡¶	1472 (53.6%; 9.29) §¶#
Non-fatal MACE	1906 (21.1%; 3.06)	1742 (25.3%; 4.26) ¶	1125 (36.8%; 7.91) ¶#	596 (17.9%; 2.38) †	782 (22.6%; 3.39) ‡¶	878 (32.0% 5.61) §¶#

Numbers of patients with values in brackets representing percentages and incidence rates per-100-person-years of follow-up (except for in-hospital mortality as incidence rates not applicable).AF, atrial fibrillation; non-fatal MACE, non-fatal major adverse cardiovascular events defined as rehospitalisation with a primary diagnosis of acute myocardial infarction or ischaemic cerebrovascular accident or congestive heart failure or revascularisation (percutaneous coronary intervention or coronary artery bypass graft); MACE, major adverse cardiovascular events defined as all-cause mortality or rehospitalisation with a primary diagnosis of acute myocardial infarction or ischaemic cerebrovascular accident or congestive heartfailure or revascularisation (percutaneous coronary intervention or coronary artery bypass graft).

∗ For the outcome endpoints, the primary diagnosis of a hospital readmission following the index cardiac valve surgery admission was used to identify non-fatal clinical outcomes including AF, acute myocardial infarction, congestive heart failure, ischaemic stroke, haemorrhagic stroke, venous thromboembolism, pulmonary embolism, deep venous thromboembolism, peripheral arterial thromboembolism, bleeding, MACE, all-cause mortality and in-hospital mortality. For AF, we separately analysed hospital readmission rates whereby either a primary or secondary diagnosis of AF was coded.

† *P* < 0.01 comparing MVSx No-AF group to AVSx No-AF group

‡ *P* < 0.01 comparing MVSx New-AF group to AVSx New-AF group

§ *P* < 0.05 comparing MVSx Prior-AF group to AVSx Prior-AF group

¶ *P* < 0.05 compared to No-AF group (within AVSx group or separately within MVSx group)

# *P* < 0.05 compared to New-AF group (within AVSx group or separately within MVSx group)

### Association of AF status on MACE and all-cause mortality in multivariable analysis

In the whole cohort and AVSx subgroup, after adjusting for differences in baseline characteristics and admission year-groups, compared to No-AF status, New-AF and Prior-AF status were independently associated with MACE and all-cause mortality (Table 3; see Supplementary Tables S3-10 for full analysis results). In the MVSx subgroup, only Prior-AF was associated with increased risk of MACE and all-cause mortality (Table 3). The subtypes of AVSx (bioprosthetic vs. mechanical valve replacement) and MVSx (repair vs. bioprosthetic or mechanical valve replacement) did not impact on the associations of AF status on MACE and all-cause mortality (Supplementary Tables S11-14). Interaction analyses between AF status (Prior-AF vs. No-AF) and the type of cardiac valve surgery showed a significant interaction on the MACE outcome (p_*interaction*_=0.03) (Supplementary Table S16). The complete series of interaction analyses is shown in Supplementary Tables S15–18.

**Table 3:** Impact of AF status on MACE and all-cause mortality in patients admitted for open-heart cardiac valve surgery

Endpoint: MACE	aHR	95% CI	*P* value
Whole cohort *			
MVSx vs. AVSx reference group	0.94	0.90–0.98	0.002
AF subgroups			
New-AF vs. No-AF reference group	1.11	1.07–1.16	< 0.001
Prior-AF vs. No-AF reference group	1.68	1.60–1.76	< 0.001
**Aortic valve surgery subgroup †**			
New-AF vs. No-AF reference group	1.14	1.08–1.19	< 0.001
Prior-AF vs. No-AF reference group	1.76	1.67–1.86	< 0.001
**Mitral valve surgery subgroup †**			
New-AF vs. No-AF reference group	1.05	0.97–1.14	0.27
Prior-AF vs. No-AF reference group	1.51	1.39–1.64	< 0.001
**Endpoint: All-cause mortality**	**aHR**	**95% CI**	**P value**
Whole cohort *			
MVSx vs. AVSx reference group	0.89	0.85–0.94	< 0.001
AF subgroups			
New-AF vs. No-AF reference group	1.13	1.08–1.18	< 0.001
Prior-AF vs. No-AF reference group	1.62	1.54–1.70	< 0.001
**Aortic valve surgery subgroup ‡**			
New-AF vs. No-AF reference group	1.16	1.10–1.22	< 0.001
Prior-AF vs. No-AF reference group	1.69	1.59–1.79	< 0.001
**Mitral valve surgery subgroup ‡**			
New-AF vs. No-AF reference group	1.06	0.96–1.17	0.24
Prior-AF vs. No-AF reference group	1.47	1.33–1.61	< 0.001

AF, atrial fibrillation; AVSx, aortic valve surgery; CABG, coronary artery bypass graft; CI, confidence interval; MACE, major adverse cardiovascular events defined as all-cause mortality or rehospitalisation with a primary diagnosis of acute myocardial infarction or ischaemic cerebrovascular accident or cardiac failure or revascularisation (percutaneous coronary intervention or coronary artery bypass graft); MVSx, mitral valve surgery; PCI, percutaneous coronary intervention.

∗ Variables included in the Cox regression multivariable analysis include type of open-heart cardiac valve surgery (aortic vs. mitral valve surgery), type of AF status, age, male sex, year of admission, referred from Emergency Department, admission to intensive care unit, comorbidities: hypertension, hyperlipidaemia, diabetes, current / ex-smoker, congestive heart failure, pulmonary embolism, deep venous thrombosis, ischaemic heart disease, prior CABG or PCI, ischaemic stroke, haemorrhagic stroke, peripheral vascular disease, malignancy, sleep apnoea, chronic kidney disease, chronic pulmonary disease, neurodegenerative disease, major bleed, concomitant CABG or PCI during index valve surgery admission, Australian Statistical Geography Standard Remoteness Area, and Index of Relative Socioeconomic Advantage and Disadvantage score (for full univariable and multivariable analysis results, see Supplementary Tables 3–5 and 8 for endpoints MACE and all-cause mortality respectively).

† Variables included in the Cox regression multivariable analysis include type of AF status, age, male sex, year of admission, referred from Emergency Department, admission to intensive care unit, comorbidities: hypertension, hyperlipidaemia, diabetes, current / ex-smoker, congestive heart failure, pulmonary embolism, deep venous thrombosis, ischaemic heart disease, prior CABG or PCI, ischaemic stroke, haemorrhagic stroke, peripheral vascular disease, malignancy, sleep apnoea, chronic kidney disease, chronic pulmonary disease, neurodegenerative disease, major bleed, concomitant CABG or PCI during index valve surgery admission, Australian Statistical Geography Standard Remoteness Area, and Index of Relative Socioeconomic Advantage and Disadvantage score (for full multivariable analysis results, see Supplementary Tables 6 and 7 for aortic and mitral valve surgery subgroups respectively).

‡ Variables included in the Cox regression multivariable analysis include type of AF status, age, male sex, year of admission, referred from Emergency Department, comorbidities: hypertension, hyperlipidaemia, diabetes, current / ex-smoker, congestive heart failure, pulmonary embolism, deep venous thrombosis, ischaemic heart disease, prior CABG or PCI, ischaemic stroke, haemorrhagic stroke, peripheral vascular disease, malignancy, sleep apnoea, chronic kidney disease, chronic pulmonary disease, neurodegenerative disease, major bleed, concomitant CABG or PCI during index valve surgery admission, Australian Statistical Geography Standard Remoteness Area, and Index of Relative Socioeconomic Advantage and Disadvantage score (for full multivariable analysis results, see Supplementary Tables 9 and 10 for aortic and mitral valve surgery subgroups respectively).

Our sensitivity analyses excluding concomitant CABG during the index cardiac valve surgery yielded similar findings in which New-AF and Prior-AF were associated with increased risk of MACE and all-cause mortality in the whole cohort and AVSx subgroup whereas Prior-AF was associated with increased risk of MACE and all-cause mortality only in the MVSx subgroup (Supplementary Tables S19-24).

### Fine-gray competing risk analyses of non-fatal cardiovascular endpoints

Competing risk analyses showed non-fatal MACE and rehospitalisation rates for AF and congestive heart failure were significantly higher in New-AF and Prior-AF compared to No-AF patients in both AVSx and MVSx subgroups while the rehospitalisation rate for ischaemic stroke was significantly higher only in the AVSx subgroup (New-AF: sHR = 1.15, 95% CI 1.01–1.32, *P* = 0.04; Prior-AF: sHR = 1.34, 95% CI 1.13–1.58, *P* < 0.001, compared to No-AF) (Table 2 for event rates and Table 4 for competing risk results). In the MVSx Prior-AF subgroup, the rehospitalisation rate for peripheral arterial thromboembolism was significantly higher (sHR = 4.56, 95% CI 1.39–14.9, *P* = 0.012). There was no significant difference in rehospitalisation rate for peripheral arterial thromboembolism in the AVSx subgroup.

**Table 4:** Risk of rehospitalisation for non-fatal clinical outcomes stratified by type of open-heart cardiac valve surgery and atrial fibrillation status during admission *

Aortic valve surgery group	No-AF group (reference)	New-AF group				Prior-AF group			
Post-discharge non-fatal CV outcomes	No. (%)	No. (%)	sHR	95% CI	*P* value	No. (%)	sHR	95% CI	*P* value
Atrial fibrillation	1622 (18.0%)	2781 (40.5%)	2.60	2.45–2.77	< 0.001	1821 (59.5%)	4.48	4.18–4.80	< 0.001
Acute myocardial infarction	461 (5.1%)	333 (4.8%)	0.89	0.77–1.03	0.13	189 (6.2%)	1.09	0.91–1.31	0.33
Congestive heart failure	1034 (11.5%)	1094 (15.9%)	1.29	1.18–1.40	< 0.001	833 (27.2%)	2.14	1.94–2.35	< 0.001
Ischaemic stroke	459 (5.1%)	417 (6.1%)	1.15	1.01–1.32	0.04	226 (7.4%)	1.34	1.13–1.58	< 0.001
Haemorrhagic stroke	163 (1.8%)	138 (2.0%)	1.13	0.89–1.42	0.31	65 (2.1%)	1.14	0.85–1.54	0.39
Pulmonary embolism	124 (1.4%)	88 (1.3%)	0.91	0.69–1.21	0.53	33 (1.1%)	0.75	0.50–1.12	0.16
Deep vein thrombosis	50 (0.6%)	33 (0.5%)	0.83	0.53–1.29	0.41	19 (0.6%)	1.00	0.59–1.70	1.00
Peripheral arterial thromboembolism	30 (0.3%)	14 (0.2%)	0.64	0.34–1.19	0.16	10 (0.3%)	1.00	0.48–2.09	0.99
Total bleeding events	2449 (27.2%)	2039 (29.7%)	1.09	1.02–1.15	0.006	1099 (35.9%)	1.31	1.22–1.41	< 0.001
Non-fatal MACE	1906 (21.1%)	1742 (25.3%)	1.15	1.07–1.22	< 0.001	1125 (36.8%)	1.69	1.57–1.83	< 0.001
**Mitral valve surgery group**	**No-AF group (reference)**	**New-AF group**				**Prior-AF group**			
**Post-discharge non-fatal CV outcomes**	**No. (%)**	**No. (%)**	**sHR**	**95% CI**	**P value**	**No. (%)**	**sHR**	**95% CI**	**P value**
Atrial fibrillation	734 (22.0%)	1473 (42.5%)	2.11	1.93–2.31	< 0.001	1657 (60.3%)	3.44	3.14–3.76	< 0.001
Acute myocardial infarction	137 (4.1%)	137 (4.0%)	0.85	0.67–1.09	0.20	119 (4.3%)	0.88	0.68–1.15	0.36
Congestive heart failure	321 (9.6%)	513 (14.8%)	1.38	1.20–1.58	< 0.001	647 (23.6%)	2.18	1.90–2.50	< 0.001
Ischaemic stroke	181 (5.4%)	185 (5.3%)	0.86	0.70–1.06	0.16	181 (6.6%)	0.99	0.80– 1.23	0.93
Haemorrhagic stroke	55 (1.7%)	72 (2.1%)	1.19	0.83–1.70	0.34	66 (2.4%)	1.29	0.89–1.86	0.18
Pulmonary embolism	50 (1.5%)	47 (1.4%)	0.89	0.58–1.36	0.58	27 (1.0%)	0.62	0.37–1.04	0.070
Deep vein thrombosis	14 (0.4%)	16 (0.5%)	1.23	0.56–2.68	0.60	11 (0.4%)	1.01	0.44– 2.35	0.97
Peripheral arterial thromboembolism	4 (0.1%)	12 (0.3%)	2.99	0.99–9.05	0.052	15 (0.5%)	4.56	1.39–14.9	0.012
Total bleeding events	794 (23.8%)	941 (27.1%)	1.09	0.99–1.20	0.079	843 (30.7%)	1.22	1.10–1.35	< 0.001
Non-fatal MACE	596 (17.9%)	782 (22.6%)	1.15	1.03–1.28	0.014	878 (32.0%)	1.65	1.48–1.84	< 0.001

AF, atrial fibrillation (primary and secondary diagnosis); CCF, congestive heart failure; CI, confidence interval; CV, cardiovascular; non-fatal MACE, non-fatal major adverse cardiovascular events defined as rehospitalisation with a primary diagnosis of acute myocardial infarction or ischaemic cerebrovascular accident or congestive heart failure or revascularisation (percutaneous coronary intervention or coronary artery bypass graft); sHR, subdistribution hazard ratio; SE, standard error.

* Fine-Gray competing risk method was used to account for the competing risk of death adjusted for AF status (No-AF vs. New-AF vs. Prior-AF), age, Charlson comorbidity index, male sex, referral from Emergency Department, admission to intensive care unit, year of admission, concomitant percutaneous coronary intervention or coronary artery bypass surgery procedure during index valve surgery admission, and Australian Statistical Geography Standard Remoteness Area.

In a separate competing risk analysis assessing patients’ subsequent risk for ischaemic stroke when CHA_2_DS_2_VASc was ≥ 1 during their index valve surgery admission, both New-AF and Prior-AF patients in the AVSx subgroup had a higher rehospitalisation rate for ischaemic stroke compared to No-AF patients (New-AF: sHR = 1.22, 95% CI 1.07–1.40, *P* = 0.004; Prior-AF: sHR = 1.43, 95% CI 1.22–1.69, *P* < 0.001 ). This however, was not observed in the MVSx cohort (Supplementary Table S25).

The rehospitalisation rate for total bleeding events was significantly higher in New-AF (sHR = 1.09, 95% CI 1.02–1.15, *P* = 0.006) and Prior-AF (sHR = 1.31, 95% CI 1.22–1.41, *P* < 0.001) compared to No-AF patients in AVSx subgroup. However, in the MVSx subgroup, only Prior-AF patients had a significantly higher rehospitalisation rate (sHR = 1.22, 95% CI 1.10–1.35, *P* < 0.001) for total bleeding events (Table 4).

## Discussion

This study examined the associations of AF status on mortality and non-fatal cardiovascular outcomes in a statewide Australian study cohort who underwent open-heart aortic or mitral valve surgery. The key findings of this study are: (1) The incidence (New-AF) and prevalence (Prior-AF) of AF in the aortic valve surgery subgroup were 36.3% and 16.1% respectively, and 36.3% and 28.8% respectively in the mitral valve surgery subgroup during patients’ index valve surgery admission; (2) During a median follow-up of 6.58 years, Prior-AF and New-AF patients had significantly higher all-cause mortality rates; (3) In the aortic valve surgery subgroup, compared to No-AF status, New-AF and Prior-AF status were independently associated with MACE and all-cause mortality. In the mitral valve surgery subgroup, only prior AF was associated with an increased risk of MACE and all-cause mortality. The subtype of AVSx and MVSx (repair, bioprosthetic or mechanical) did not change the impact of AF status on MACE and all-cause mortality; (4) Competing risk analyses showed non-fatal MACE and rehospitalisation rates for AF and congestive heart failure were significantly higher in New-AF and Prior-AF compared to No-AF patients in both AVSx and MVSx subgroups while the rehospitalisation rate for ischaemic stroke was significantly higher only in the AVSx subgroup.

### Incidence of POAF and association with all-cause mortality and MACE

The overall AF incidence rate of 36.3% in our study is consistent with rates reported in other Western countries such as United States (33.7%), Canada (36.6%), Europe (34%), but higher than South America (17.4%) and Asia (15.7%), and lower than Middle East (41.6%), according to a multi-centre study by Matthew et al. [21] .

A population-based observational study by Kalra et al. (*n* = 122 765) found that the incidence of new-onset AF in aortic valve replacement (AVR) hospitalisations was 50.1% [22] while a cohort study by Aggarwal et al. (*n* = 504) found that the incidence of new-onset AF in mitral valve surgery was 60.2% [23]. Another prospective multicentre cohort study by Herrmann et al. (*n* = 198) showed that the cumulative incidence of new-onset AF within the first year after CABG was 48% [24]. In contrast, our study had a lower incidence rate of AF (36.3%) in patients who had undergone AVR. The difference in incidence could be due to different methods of detecting the outcome of new-onset AF. Both Kalra and Aggarwal et al. used telemetry and electrocardiogram [22, 23] while Herrmann et al. used insertable cardiac monitors [24] unlike our study which relied on administrative data (ICD-10AM codes) which may have coding errors leading to potentially either an underestimate or overestimate of the incidence rates of POAF.

A systematic review and meta-analysis by Kaur et al. consisting of 8 single-centre prospective cohort studies (*n* = 185) showed that the recurrence of new-onset POAF after cardiac surgery detected by implantable loop recorders was 35.5% at 18 months [25]. Our study had higher recurrence rates of POAF ranging from 40 to 60% probably due to a longer median follow-up time of 6.58 years.

Consistent with our study, meta-analyses have shown POAF is associated with increased short-term [10] and long-term [9, 10] mortality risk. The overall in-hospital mortality rate of 2.5% is similar to the in-hospital mortality rate of 2–4% in a meta-analysis which included studies that utilised different methods to detect AF [26]. The meta-analysis by Woldendorp et al. however, found POAF was not associated with higher mortality in the cardiac valve surgery subgroups compared to the post-CABG, post-heart failure surgery, post-combined CABG, post-valve surgery subgroups and other heterogenous cardiac procedures [10]. There was a large degree of heterogeneity in the included studies and the definitions of POAF in the various studies were highly variable, including time-based definitions varying from greater than 30 s to 1 h, while other studies defined POAF as any episode that resulted in symptoms or only those that required treatment (ranging from electrolyte replacement to antiarrhythmic drugs).

Contrary to our study findings, a study by Almassi et al. (*n* = 2203) showed no 5-year risk-adjusted outcome differences, including MACE, in patients with and without POAF (OR = 1.06, 99%CI = 0.80–1.41, *P* = 0.58). However, this study included only patients after CABG [27]. In addition, a study by Akintoye et al. (*n* = 1462) found a prior history of AF did not independently predict MACE in patients who underwent open-heart cardiac surgery. Unlike our study, this study did not examine the relationship between new-onset AF and MACE in open-heart cardiac surgery patients [28].

### Comparison between outcomes in patients who underwent AVSx versus MVSx

The association of AF with all-cause mortality concurs with the study by Giered et al. which showed POAF was significantly associated with reduced long-term survival (75.9 ± 1.6% vs. 82.6 ± 1.2% at 6 years, *P* < 0.001) following aortic valve replacement (AVR) but not after MV replacement or repair (84.4 ± 2.4% vs. 87.8 ± 2.0%, *P* = 0.40). However, this was a single-centre study with a much smaller cohort (*n* = 2986) compared to ours [13]. Our study further extends the findings of this study by examining the associations of AF status on MACE and other non-fatal outcomes including atrial fibrillation, myocardial infarction, congestive heart failure, stroke, venous thromboembolism, peripheral arterial thromboembolism, haemorrhagic stroke and bleeding. The findings by Giered et al. were attributed to underlying factors involved in the pathogenesis of POAF in each type of cardiac valve surgery. In this study, patients with AVR were mainly patients with severe aortic stenosis which shares pathophysiological similarities with coronary artery disease. In contrast, POAF might be related to the preoperative features of mitral valve disease (left atrial dilatation and hypertension), which are at least partially corrected by surgery [13]. These hypotheses will need to be confirmed in future mechanistic studies.

### Rehospitalisation rates for non-fatal cardiovascular outcomes

Rehospitalisation rates for AF and congestive heart failure were significantly higher in New-AF and Prior-AF compared to No-AF patients in both AVSx and MVSx subgroups. This mirrors the findings of other studies. A study by Bianco et al. found that POAF was associated with a higher incidence of AF on follow-up (11.74% vs. 4.75%; *P* < 0.001) and a higher rate of heart failure–specific readmission (HR = 1.14, 95%CI = 1.04–1.26, *P* = 0.01) [29]. Goyal et al. also found POAF was associated with incident HF hospitalisation (HR = 1.33, 95%CI = 1.25–1.41) [30]. However, these studies did not account for the competing risk of death. These studies also did not seek to differentiate the impact of new vs. prior-AF on outcomes.

Several studies have shown an increased risk of stroke in patients with POAF which are consistent with our findings [10, 14, 31]. It is important to note that in our study, the rehospitalisation rate for ischaemic stroke was significantly higher only in the AVSx subgroup. This differs from the study by Bucerius et al. (*n* = 16184) which reported patients who underwent MVSx had significantly more stroke events than those who had AVSx [14]. However, it was a single-centre study with a heterogenous design that included different types of cardiac valve procedures including multiple valve surgery and CABG [14].

Our study is the first to report the rehospitalisation rate for peripheral arterial thromboembolism was significantly higher only in Prior-AF patients in the MVSx subgroup. An observational study by Butt et al. found that new-onset POAF after left-sided heart valve surgery was associated with a similar long-term risk of thromboembolism as nonvalvular AF, but thromboembolism in this study was defined as a composite endpoint consisting of ischaemic stroke, transient cerebral ischaemia and thrombosis or embolism in peripheral arteries [32].

While the rehospitalisation rate for bleeding was significantly higher in New-AF and Prior-AF compared to No-AF patients in the AVSx subgroup, it was significantly higher only in Prior-AF patients in the MVSx subgroup. The increased bleeding risk in patients who had prior AF and mitral valve pathology could be attributed to the need for long-term anticoagulation to prevent cardioembolic stroke in the setting of valvular AF. There was also a numerically higher rate of bleeding events for the New-AF MVSx subgroup though it did not reach significance in competing risk analysis.

### Strengths and limitations

Our study has the following strengths. This is the first large-scale Australian study examining the impact of AF on specific clinical outcomes in patients after hospitalisation for open-heart cardiac valve surgery. It includes a large statewide cohort of all patients hospitalised for cardiac valve surgery linked to a death registry, linkage to a statewide hospital admission database with adequate estimation of rehospitalisation rates, availability of a control group without AF, stratification of AF status into Prior-AF and New-AF and an adequate long-term follow-up period with a median follow-up time of at least 6 years. Patients who had multivalve surgery were excluded to facilitate clear comparisons between aortic valve versus mitral valve surgery.

However, there are several limitations to note. Firstly, this is a retrospective cohort study. Data variables were derived from an administrative dataset with potential coding errors. Post-discharge clinical events may have been underestimated as we were not able to capture events not requiring hospitalisation. As a consequence, our baseline AF status may be an underestimate due to not capturing AF episodes that occurred in the community that did not require hospitalisation. We did not have access to cause-specific death records and thus was limited to all-cause mortality analysis. There was also no data on medications, including the use of anticoagulation therapies. Future studies should examine if some of the outcome associations observed in the present study could be accounted for by the use of anticoagulation. The timing of in-hospital AF in relation to the timing of the cardiac valve surgery was also unknown. There was also no specific timing of AF in the community as AF was defined based on AF hospitalisation. It was also beyond the scope of this study to further differentiate the types of valvular surgery performed (e.g. repair vs. replacement, mechanical vs. bioprosthetic) and future studies should examine if there are differential incidence and impact of AF for these subgroups. At the very least, this present study serves as a benchmark for the rates of AF and other adverse clinical outcomes in patients undergoing AVSx and MVSx.

## Conclusions

Patients who had undergone cardiac valve surgery are at high risk of all-cause mortality and other adverse clinical outcomes, with the type of cardiac valve surgery and AF status being associated with differences in clinical outcomes. Drivers behind this difference should be explored further in future studies. This will facilitate the development of prevention and treatment strategies which are tailored accordingly to different surgical patient groups to minimise their risk of adverse outcomes. In addition, future studies that include anticoagulation and therapies such as AF ablation should be conducted to determine if such treatments targeting AF alter the prognosis of these surgical patient cohorts.

## Supplementary Information

Below is the link to the electronic supplementary material.

Supplementary Material 1: Additional file 1: Table S1-S25, Figure S1

## Data Availability

The New South Wales Population and Health Services Research Ethics Committee prohibits the authors from making the minimal data set publicly available. Interested researchers may contact the Ethics coordinator (ethics@cancerinstitute.org.au) to seek permission to access the data; data will then be made available upon request to all interested researchers who receive approval from the New South Wales Population and Health Services Research Ethics Committee.

## Abbreviations

AF: Atrial fibrillation
APDC: Admitted-Patient-Data-Collection
ASGS-RA: Australian Statistical Geography Standard – Remoteness Area
AVSx: Aortic valve surgery
CABG: Coronary artery bypass graft
CHeReL: Centre for Health Record Linkage
ICD-10AM: International Statistical Classification of Diseases and Related Health Problems, Tenth Revision, Australian Modification
ICD: Implantable Cardioverter-Defibrillator
IQR: Interquartile range
IRSAD score: Index of relative socio-economic advantage and disadvantage score
MVSx: Mitral valve surgery
PCI: Percutaneous coronary intervention
PHSREC: Population and Health Services Research Ethics Committee
POAF: Postoperative atrial fibrillation
PPM: Permanent pacemaker
SD: Standard deviation
