# Supplementary material for: Impact of atrial fibrillation on clinical outcomes in 28,492 patients following open-heart cardiac valve surgery: A statewide population-linkage study
Source: BMC Med. 2026 May 27;24:508. doi: 10.1186/s12916-026-04953-2 (PMC13595643; doi:10.1186/s12916-026-04953-2)
Supplement: Supplementary file 1 [file 12916_2026_4953_MOESM1_ESM.docx]

**Supplemental Material**

Ne et al; Impact of AF status on cardiac valve surgery patients

**Supplementary Table S1. Study comorbidities** **International Statistical Classification of Diseases and Related Health Problems, Tenth Revision, Australian Modification (ICD-10AM) codes and Australian Classification of Health Interventions (ACHI) procedural codes**

| **No.** | **Morbidity *** | **ICD-10AM codes** |
| --- | --- | --- |
| 1 | Pulmonary embolism (PE) | I26, I26.0, I26.9 |
| 2 | Deep vein thrombosis (DVT) | I80, I80.0, I80.1, I80.2, I80.3, I80.8, I80.9, I81, I82, I82.0, I82.1, I82.2, I82.3, I82.8, I82.9 |
| 3 | Venous thromboembolism (VTE) (defined PE and/or DVT) | I26, I26.0, I26.9, I80, I80.0, I80.1, I80.2, I80.3, I80.8, I80.9, I81, I82, I82.0, I82.1, I82.2, I82.3, I82.8, I82.9 |
| 4 | Atrial fibrillation and/or flutter | I48, I48.0, I48.1, I48.2, I48.3, I48.4, I48.9 |
| 5 | Acute myocardial infarction | I21, I21.0, I21.1, I21.2, I21.3, I21.4, I21.9, I22, I22.0, I22.1, I22.8, I22.9 |
| 6 | Ischaemic heart disease | I20, I20.0, I20.1, I20.8, I20.9, I21, I21.0, I21.1, I21.2, I21.3, I21.4, I21.9, I22, I22.0, I22.1, I22.8, I22.9, I23, I23.0, I23.1, I23.2, I23.3, I23.4, I23.5, I23.6, I23.8, I24, I24.0, I24.1, I24.8, I24.9, I25, I25.0, I25.1, I25.10, I25.11, I25.12, I25.13, I25.2, I25.3, I25.4, I25.5, I25.6, I25.8, I25.9 |
| 7 | Prior coronary artery bypass graft (CABG) | Z95.1 |
| 8 | Prior percutaneous coronary intervention (PCI) | Z95.5 |
| 10 | Congestive heart failure | I42, I42.0, I42.1, I42.2, I42.3, I42.4, I42.5, I42.6, I42.7, I42.8, I42.9, I43, I43.0, I43.1, I43.2, I43.8, I50, I50.0, I50.1, I50.9, I11.0, I13.0, I13.2 |
| 11 | Valvular heart disease | I05, I05.0, I05.1, I05.2, I05.8, I05.9, I06, I06.0, I06.1, I06.2, I06.8, I06.9, I07, I07.0, I07.1, I07.2, I07.8, I07.9, I08, I08.0, I08.1, I08.2, I08.3, I08.8, I08.9, I09.1, I09.8, I34, I34.0, I34.1, I34.2, I34.8, I34.9, I35, I35.0, I35.1, I35.2, I35.8, I35.9, I36, I36.0, I36.1, I36.2, I36.8, I36.9, I37, 37.0, I37.1, I37.2, I37.8, I37.9, I38, I39, I39.0, I39.1, I39.2, I39.3, I39.4, I39.8, Q22, Q22.0, Q22.1, Q22.2, Q22.3, Q22.30, Q22.31, Q22.39, Q22.4, Q22.41, Q22.42, Q22.5, Q22.6, Q22.8, Q22.81, Q22.82, Q22.89, Q22.9, Q23, Q23.0, Q23.01, Q23.02, Q23.1, Q23.2, Q23.21, Q23.22, Q23.3, Q23.4, Q23.8, Q23.81, Q23.82, Q23.83, Q23.89, Q23.9, Q24.87 |
| 12 | Peripheral vascular disease | E09.5, E09.51, E09.52, E10.51, E10.52, E11.51, E11.52, E13.51, E13.52, E14.51, E14.52, I70, I70.0, I70.1, I70.2, I70.20, I70.21, I70.22, I70.23, I70.24, I70.8, I70.9, I71, I71.0, I71.00, I71.01, I71.02, I71.03, I71.1, I71.2, I71.3, I71.4, I71.5, I71.6, I71.8, I71.9, I72, I72.0 I72.1, I72.2, I72.3, I72.4, I72.5, I72.6, I72.8, I72.9, I73, I73.0, I73.1, I73.8, I73.9, I74, I74.0, I74.1, I74.2, I74.3, I74.4, I74.5, I74.8, I74.9, I77, I77.0, I77.1, I77.2, I77.3, I77.4, I77.5, I77.6, I77.8, I77.9, I78, I78.0, I78.1, I78.8, I78.9, I79, I79.0, I79.1, I79.2, I79.8 |
| 13 | Total stroke | G45, G45.0, G45.1, G45.2, G45.3, G45.4, G45.8, G45.9, G46, G46.0, G46.1, G46.2, G46.3, G46.4, G46.5, G46.6, G46.7, G46.8, I60, I60.0, I60.1, I60.2, I60.3, I60.4, I60.5, I60.6, I60.7, I60.8, I60.9, I61, I61.0, I61.1, I61.2, I61.3, I61.4, I61.5, I61.6, I61.8, I61.9, I62, I62.0, I62.1, I62.9, I63, I63.0, I63.1, I63.2, I63.3, I63.4, I63.5, I63.6, I63.8, I63.9, I64   - TIA: G45, G45.0, G45.1, G45.2, G45.3, G45.4, G45.8, G45.9 - Vascular syndromes of brain in cerebrovascular diseases: G46, G46.0, G46.1, G46.2, G46.3, G46.4, G46.5, G46.6, G46.7, G46.8 - Haemorrhagic stroke: I60, I60.0, I60.1, I60.2, I60.3, I60.4, I60.5, I60.6, I60.7, I60.8, I60.9, I61, I61.0, I61.1, I61.2, I61.3, I61.4, I61.5, I61.6, I61.8, I61.9, I62, I62.0, I62.1, I62.9 - Ischaemic stroke: I63, I63.0, I63.1, I63.2, I63.3, I63.4, I63.5, I63.6, I63.8, I63.9, I64 |
| 20 | Hypertension | I10, I11, I11.0, I11.9, I12, I12.0, I12.9, I13, I13.0, I13.1, I13.2, I13.9, I15, I15.0, I15.1, I15.2, I15.8, I15.9 |
| 21 | Hyperlipidaemia | E78, E78.0, E78.1, E78.2, E78.3, E78.4, E78.5, E78.6, E78.8, E78.9 |
| 22 | Diabetes | E09, E09.2, E09.21, E09.29, E09.3, E09.31, E09.32, E09.4, E09.40, E09.42, E09.5, E09.51, E09.52, E09.7, E09.71, E09.72, E09.8, E09.9, E10, E10.0, E10.01, E10.02, E10.1, E10.11, E10.12, E10.13, E10.14, E10.15, E10.16, E10.2, E10.21, E10.22, E10.29, E10.3, E10.31, E10.32, E10.33, E10.34, E10.35, E10.36, E10.39, E10.4, E10.40, E10.41, E10.42, E10.43, E10.49, E10.5, E10.51, E10.52, E10.53, E10.6, E10.61, E10.62, E10.63, E10.64, E10.65, E10.69, E10.7, E10.71, E10.73, E10.8, E10.9, E11, E11.0, E11.01, E11.02, E11.1, E11.11, E11.12, E11.13, E11.14, E11.15, E11.16, E11.2, E11.21, E11.22, E11.29, E11.3, E11.31, E11.32, E11.33, E11.34, E11.35, E11.36, E11.39, E11.4, E11.40, E11.41, E11.42, E11.43, E11.49, E11.5, E11.51, E11.52, E11.53, E11.6, E11.61, E11.62, E11.63, E11.64, E11.65, E11.69, E11.7, E11.71, E11.72, E11.73, E11.8, E11.9, E13, E13.0, E13.01, E13.02, E13.1, E13.11, E13.12, E13.13, E13.14, E13.15, E13.16, E13.2, E13.21, E13.22, E13.29, E13.3, E13.31, E13.32, E13.33, E13.34, E13.35, E13.36, E13.39, E13.4, E13.40, E13.41, E13.42, E13.43, E13.49, E13.5, E13.51, E13.52, E13.53, E13.6, E13.61, E13.62, E13.63, E13.64, E13.65, E13.69, E13.7, E13.71, E13.72, E13.73, E13.8, E13.9, E14, E14.0, E14.01, E14.02, E14.1, E14.11, E14.12, E14.13, E14.14, E14.15, E14.16, E14.2, E14.21, E14.22, E14.29, E14.3, E14.31, E14.32, E14.33, E14.34, E14.35, E14.36, E14.39, E14.4, E14.40, E14.41, E14.42, E14.43, E14.49, E14.5, E14.51, E14.52, E14.53, E14.6, E14.61, E14.62, E14.63, E14.64, E14.65, E14.69, E14.7, E14.71, E14.72, E14.73, E14.8, E14.9, Z92.22 |
| 25 | Current/ex-smoker | F17, Z72.0, Z86.43 |
| 29 | Systemic connective tissue disease | M30, M30.0, M30.1, M30.2, M30.3, M30.8, M31, M31.0, M31.1, M31.2, M31.3, M31.4, M31.5, M31.6, M31.7, M31.8, M31.9, M32, M32.0, M32.1, M32.8, M32.9, M33, M33.0, M33.1, M33.2. M33.9, M34, M34.0, M34.1, M34.2, M34.8, M34.9, M35, M35.0, M35.1, M35.2, M35.3, M35.4, M35.5, M35.6, M35.7, M35.8, M35.9, M36, M36.0, M36.1, M36.2, M36.3, M36.4, M36.8 |
| 30 | Chronic pulmonary disease | E84.0, E84.0, E84.1, E84.8, E84.9, J40, J41, J41.0, J41.1, J41.8, J42, J43, J43.0, J43.1, J43.2, J43.8, J43.9, J44, J44.0, J44.1, J44.8, J44.9, J45, J45.0, J45.1, J45.8, J45.9, J46, J47, J60, J61, J62, J62.0, J62.8, J63, J63.0, J63.1, J63.2, J63.3, J63.4, J63.5, J63.8, J64, J65, J66, J66.0, J66.1, J66.2, J66.8, J67, J67.0, J67.1, J67.2, J67.3, J67.4, J67.5, J67.6, J67.7, J67.8, J67.9, J68, J68.0, J68.1, J68.2, J68.3, J68.4, J68.8, J68.9, J70, J70.0, J70.1, J70.2, J70.3, J70.4, J70.8, J70.9, J82, J84, J84.0, J84.1, J84.8. J84.9, J99, J99.1, J99.8 |
| 32 | Malignancy | C00-C96, D00-D09 |
| 33 | Sleep apnoea | G47.3, G47.30, G47.31, G47.32, G47.33, G47.39, G47.4, G47.8, G47.9 |
| 35 | Chronic kidney disease | N18, N18.1, N18.2, N18.3, N18.4, N18.5, N18.9, N19 |
| 37 | Dementia | F00, F00.0, F00.1, F00.2, F00.9, F01, F01.0, F01.1, F01.2, F01.3, F01.8, F01.9, F02, F02.0, F02.1, F02.2, F02.3, F02.4, F02.8, F03 |
| 40 | Neurodegenerative diseases (defined as dementia, central nervous systemic atrophies, Parkinson’s disease, basal ganglia degeneration and/or nervous systemic degenerative diseases) | F00, F00.0, F00.1, F00.2, F00.9, F01, F01.0, F01.1, F01.2, F01.3, F01.8, F01.9, F02, F02.0, F02.1, F02.2, F02.3, F02.4, F02.8, F03, G10, G11, G11.0, G11.1, G11.2, G11.3, G11.4, G11.8, G11.9, G12, G12.0, G12.1, G12.2, G12.8, G12.9, G13, G13.0, G13.1, G13.2, G13.8, G14, G20, G23, G23.0, G23.1, G23.2, G23.8, G23.9, G30, G30.0, G30.1, G30.8, G30.9, G31, G31.0, G31.1, G31.2, G31.3, G31.8, G31.9 |
| 41 | Peptic ulcer disease | K25, K26, K27, K28 |
| 42 | Liver disease – mild | K70.0, K70.1, K70.2, K70.9, K71.0, K71.1, K71.2, K71.3, K71.4, K71.5, K71.6, K71.8, K71.9, K73, K73.0, K73.1, K73.2, K73.8, K73.9, K75, K75.0, K75.1, K75.2, K75.3, K75.4, K75.8, K75.9, K76, K76.0, K76.1, K76.2, K76.3, K76.4, K76.5, K76.6, K76.7, K76.8, K76.9, K77, K77.0, K77.8 |
| 43 | Liver disease – mod-severe | I82.0, K70.3, K70.4, K71.7, K72, K72.0, K72.1, K72.9, K74, K74.0, K74.1, K74.2, K74.3, K74.4, K74.5, K74.6 |
| 44 | Chronic kidney disease – mod-severe | N18.3, N18.4, N18.5 |
| 45 | Diabetes with organ damage | E09.21, E09.29, E09.31, E09.32, E09.40, E09.42, E09.51, E09.52, E09.71, E09.72, E09.8, E10.21, E10.22, E10.29, E10.31, E10.32, E10.33, E10.34, E10.35, E10.36, E10.39, E10.40, E10.41, E10.42, E10.43, E10.49, E10.51, E10.52, E10.53, E10.61, E10.62, E10.63, E10.69, E10.71, E10.73, E10.8, E11.21, E11.22, E11.29, E11.31, E11.32, E11.33, E11.34, E11.35, E11.36, E11.39, E11.40, E11.41, E11.42, E11.43, E11.49, E11.51, E11.52, E11.53, E11.61, E11.62, E11.63, E11.69, E11.71, E11.72, E11.73, E11.8, E13.21, E13.22, E13.29, E13.31, E13.32, E13.33, E13.34, E13.35, E13.36, E13.39, E13.40, E13.41, E13.42, E13.43, E13.49, E13.51, E13.52, E13.53, E13.61, E13.62, E13.63, E13.69, E13.71, E13.72, E13.73, E13.8, E14.21, E14.22, E14.29, E14.31, E14.32, E14.33, E14.34, E14.35, E14.36, E14.39, E14.40, E14.41, E14.42, E14.43, E14.49, E14.51, E14.52, E14.53, E14.61, E14.62, E14.63, E14.69, E14.71, E14.72, E14.73, E14.8 |
| 46 | Lymphoma | C81, C81.0, C81.1, C81.2, C81.3, C81.4, C81.7, C81.9, C82, C82.0, C82.1, C82.2, C82.3, C82.4, C82.5, C82.6, C82.7, C82.9, C83, C83.0, C83.1, C83.3, C83.5, C83.7, C83.8, C83.9, C84, C84.0, C84.1, C84.4, C84.5, C84.6, C84.7, C84.8, C84.9, C85, C85.1, C85.2, C85.7, C85.9, C86, C86.0, C86.1, C86.2, C86.3, C86.4, C86.5, C86.6, C88, C88.0, C88.2, C88.3, C88.4, C88.7, C88.9 |
| 47 | Leukaemia | C90, C90.0, C90.1, C90.2, C90.3, C91, C91.0, C91.1, C91.3, C91.4, C91.5, C91.6, C91.7, C91.8, C91.9, C92, C92.0, C92.1, C92.2, C92.3, C92.4, C92.5, C92.6, C92.7, C92.8, C92.9, C93, C93.0, C93.1, C93.3, C93.7, C93.9, C94, C94.0, C94.2, C94.3, C94.4, C94.6, C94.7, C95, C95.0, C95.1, C95.7, C95.9, C96, C96.0, C96.2, C96.4, C96.5, C96.6, C96.7, C96.8, C96.9 |
| 48 | Metastatic solid tumour | C76, C76.0, C76.1, C76.2, C76.3, C76.4, C76.5, C76.7, C76.8, C77, C77.0, C77.1, C77.2, C77.3, C77.4, C77.5, C77.8, C77.9, C78, C78.0, C78.1, C78.2, C78.3, C78.4, C78.5, C78.6, C78.7, C78.8, C79, C79.0, C79.1, C79.2, C79.3, C79.4, C79.5, C79.6, C79.7, C79.8, C79.81, C79.82, C79.88, C79.9, C80, C80.0, C80.9 |
| 49 | Acquired immunodeficiency syndrome | B20, B21, B22, B23, B23.0, B23.8, B24 |
| 50 | Hemiplegia | G81, G81.0, G81.1, G81.9, G82, G82.0, G82.1, G82.2, G82.3, G82.4, G82.5 |
| 51 | Any tumour/malignancy excluding lymphoma and/or leukaemia | C00-C80, D00-D09 |
| 54 | Peripheral arterial thromboembolism | I74, I74.0, I74.1, I74.2, I74.3, I74.4, I74.5, I74.8, I74.9 |
| 55 | Major bleed | D62, H11.3, H35.6, H43.1, J94.2, N02, N95.0, R04, R04.0, R04.1, R04.2, R04.8, R04.9, R31, R58 |
| 56 | Total Bleed Events †   - Total bleeding events (include both primary and secondary diagnoses) were based on ICD-10AM (Australian Modification) codes, adapted from bleeding events based on ICD-10 codes as described by Lip G. et al. Oral anticoagulation, aspirin, or no therapy in patients with nonvalvular AF with 0 or 1 stroke risk factor based on the CHA2DS2-VASc Score. JACC 2015;65: 1385-94. | - Haemorrhage CVA only: I60, I60.0, I60.1, I60.2, I60.3, I60.4, I60.5, I60.6, I60.7, I60.8, I60.9, I61, I61.0, I61.1, I61.2, I61.3, I61.4, I61.5, I61.6, I61.8, I61.9, I62, I62.0, I62.1, I62.9 - Major bleeding: D62, H11.3, H35.6, H43.1, J94.2, N02, N95.0, R04, R04.0, R04.1, R04.2, R04.8, R04.9, R31, R58 - Gastrointestinal bleeding: K22.6, K22.8, K25.0, K25.2, K25.4, K25.6, K26.0, K26.2, K26.4, K27.0, K27.2, K27.4, K27.6, K28.0, K28.2, K28.4, K28.6, K29.0, K29.21, K29.31, K29.41, K29.51, K29.61, K29.71, K29.81, K29.91, K31.82, K55.22, K57.01, K57.03, K57.11, K57.13, K57.21, K57.23, K57.31, K57.33, K57.41, K57.43, K57.51, K57.53, K57.81, K57.83, K57.91, K57.93. K62.5, K66.1, K92.0, K92.1, K92.2 - Traumatic intracranial haemorrhage: S06.23, S06.33, S06.34, S06.4, S06.5, S06.6, S06.8 |

- To calculate the Charlson Comorbidity Index (CCI) score, without age adjustment, for individual patient during a particular admission of interest, use the following morbidity item numbers with their corresponding ICD-10AM codes to derive the particular patient’s CCI score:
  a) 1 score for each morbidity item – 5, 10, 12, 13, 22, 29, 30, 37, 41, 42
  b) 2 score for each morbidity item – 44, 45, 46, 47, 50, 51
  c) 3 score for morbidity item – 43
  d) 6 score for morbidity item – 48, 49

| **No.** | **ACHI procedures** | **ACHI procedural codes** |
| --- | --- | --- |
| 1 | CABG | 38497-00, 38497-01, 38497-02, 38497-03, 38497-04, 38497-05, 38497-06, 38497-07, 38500-00, 38500-01, 38500-02, 38500-03, 38500-04, 38500-05, 38503-00, 38503-01, 38503-02, 38503-03, 38503-04, 38503-05, 90201-00, 90201-01, 90201-02, 90201-03 |
| 2 | PCI | 35304-00, 35305-00, 35310-00, 35310-01, 35310-02, 38300-00, 38303-00, 38306-00, 38306-01, 38306-02 |
| 3 | Permanent pacemaker implantation | 38353-00, 38281-00, 38281-01, 38281-02, 38281-03, 38281-04, 38281-05, 38281-06, 38281-07, 38281-08, 38281-09, 38281-10 |
| 4 | Implantable cardioverter defibrillator | 38393-00 |
| 5 | Open-heart aortic valve surgery | 38456-10, 38456-15, 38475-02, 38477-02, 38480-00, 38481-00, 38483-00, 38488-00, 38488-01, 38489-00, 38489-01, 38653-04 |
| 6 | Open-heart mitral valve surgery | 38456-16, 38475-00, 38477-00, 38480-01, 38481-01, 38485-00, 38485-01, 38487-00, 38488-02, 38488-03, 38489-02, 38653-05 |

**Supplementary Table S2. Baseline characteristics of study cohort stratified by type of open-heart cardiac valve surgery and atrial fibrillation status during admission***

| **Parameters** | **Aortic valve surgery (N=18949)** | | | **Mitral valve surgery (N=9543)** | | | | |
| --- | --- | --- | --- | --- | --- | --- | --- | --- |
|  | **No-AF group**  **(N=9017, 47.6%)** | **New-AF group**  **(N=6874, 36.3%)** | **Prior-AF group**  **(N=3058, 16.1%)** | **No-AF group**  **(N=3330, 34.9%)** | | **New-AF group**  **(N=3466, 36.3%)** | | **Prior-AF group**  **(N=2747, 28.8%)** |
| Age, years | 70.6 (61.2–77.6) | 74.5 (68.1–80.1) ¶ | 76.8 (70.6–81.9) ¶# | 61.8 (51.4–71.0) † | | 69.0 (60.7–76.2) ‡¶ | | 71.3 (63.6–77.7) §¶# |
| Males | 6118 (67.8%) | 4627 (67.3%) | 2100 (68.7%) | 2184 (65.6%) † | | 2122 (61.2%) ‡¶ | | 1534 (55.8%) §¶# |
| Length of stay, days | 9.0 (8.0–13.0) | 12.0 (9.0–17.0) ¶ | 13.0 (9.0–20.0) ¶# | 9.0 (8.0–14.0) | | 12.0 (9.0–18.0) ¶ | | 12.0 (9.0–18.0) §¶ |
| Referred from ED | 726 (8.1%) | 540 (7.9%) | 267 (8.7%) | 374 (11.2%) † | | 386 (11.1%) ‡ | | 175 (6.4%) §¶# |
| ICU admission | 8065 (89.4%) | 6165 (89.7%) | 2762 (90.3%) | 2950 (88.6%) | | 3062 (88.3%) ‡ | | 2404 (87.5%) § |
| IRSAD score | 992.6 (936.3–1057.7) | 990.5 (935.1–1 055.5) | 989.8 (933.2–1 063.1) | 1003.3 (941.8–1073.9) † | | 1006.5 (942.8– 1073.9) ‡ | | 992.6 (936.2–1065.3) ¶# |
| ASGSRA status |  |  |  |  | |  | |  |
| Metropolitan | 6401 (71.0%) | 4656 (67.7%) ¶ | 2115 (69.2%) ¶ | 2512 (75.4%) | † | 2560 (73.9%) | ‡ | 1941 (70.7%) ¶# |
| Regional | 2136 (23.7%) | 1763 (25.6%) ¶ | 738 (24.1%) ¶ | 650 (19.5%) |  | 705 (20.3%) |  | 632 (23.0%) ¶# |
| Remote | 474 (5.3%) | 453 (6.6%) ¶ | 205 (6.7%) ¶ | 166 (5.0%) |  | 197 (5.7%) |  | 174 (6.3%) ¶# |
| **Cardiac risk factors** |  |  |  |  | |  | |  |
| Hypertension | 3813 (42.3%) | 3208 (46.7%) ¶ | 1429 (46.7%) ¶ | 962 (28.9%) † | | 1129 (32.6%) ‡¶ | | 889 (32.4%) §¶ |
| Hyperlipidaemia | 982 (10.9%) | 733 (10.7%) | 295 (9.6%) | 268 (8.0%) † | | 243 (7.0%) ‡ | | 179 (6.5%) §¶ |
| Diabetes | 2131 (23.6%) | 1665 (24.2%) | 847 (27.7%) ¶# | 379 (11.4%) † | | 421 (12.1%) ‡ | | 421 (15.3%) §¶# |
| Current/ex-smoker | 4173 (46.3%) | 3151 (45.8%) | 1325 (43.3%) ¶# | 1311 (39.4%) † | | 1352 (39.0%) ‡ | | 1 057 (38.5%) § |
| **Co-morbidities** |  |  |  |  | |  | |  |
| CHF | 816 (9.0%) | 913 (13.3%) ¶ | 628 (20.5%) ¶# | 454 (13.6%) † | | 617 (17.8%) ‡¶ | | 642 (23.4%) §¶# |
| Venous thromboembolism   - DVT - PE | 69 (0.8%)  52 (0.6%)  21 (0.2%) | 96 (1.4%) ¶  83 (1.2%) ¶  18 (0.3%) | 45 (1.5%) ¶  40 (1.3%) ¶  8 (0.3%) | 41 (1.2%) †  33 (1.0%) †  13 (0.4%) | | 56 (1.6%)  49 (1.4%)  10 (0.3%) | | 38 (1.4%)  28 (1.0%)  12 (0.4%) |
| Ischaemic heart disease | 3554 (39.4%) | 3166 (46.1%) ¶ | 1406 (46.0%) ¶ | 936 (28.1%) † | | 1153 (33.3%) ‡¶ | | 881 (32.1%) §¶ |
| Prior CABG or PCI | 655 (7.3%) | 460 (6.7%) | 312 (10.2%) ¶# | 130 (3.9%) † | | 112 (3.2%) ‡ | | 147 (5.4%) §¶# |
| Ischaemic stroke | 122 (1.4%) | 138 (2.0%) ¶ | 83 (2.7%) ¶# | 76 (2.3%) † | | 91 (2.6%) ‡ | | 61 (2.2%) |
| Haemorrhagic stroke | 14 (0.2%) | 17 (0.2%) | 7 (0.2%) | 16 (0.5%) † | | 8 (0.2%) ¶ | | 7 (0.3%) |
| Peripheral vascular disease | 753 (8.4%) | 624 (9.1%) | 318 (10.4%) ¶# | 159 (4.8%) † | | 160 (4.6%) ‡ | | 139 (5.1%) § |
| Malignancy | 59 (0.7%) | 47 (0.7%) | 18 (0.6%) | 15 (0.5%) | | 16 (0.5%) | | 23 (0.8%) |
| Sleep apnoea | 184 (2.0%) | 125 (1.8%) | 79 (2.6%) # | 45 (1.4%) † | | 40 (1.2%) ‡ | | 51 (1.9%) # |
| Chronic kidney disease | 496 (5.5%) | 579 (8.4%) ¶ | 371 (12.1%) ¶# | 135 (4.1%) † | | 200 (5.8%) ‡¶ | | 204 (7.4%) §¶# |
| Chronic pulmonary disease | 407 (4.5%) | 395 (5.7%) ¶ | 237 (7.8%) ¶# | 109 (3.3%) † | | 164 (4.7%) ‡¶ | | 159 (5.8%) §¶ |
| Neurodegenerative disease | 46 (0.5%) | 41 (0.6%) | 27 (0.9%) | 4 (0.1%) † | | 11 (0.3%) | | 12 (0.4%) §¶ |
| Major bleed | 607 (6.7%) | 633 (9.2%) ¶ | 258 (8.4%) ¶ | 207 (6.2%) | | 315 (9.1%) ¶ | | 206 (7.5%) ¶# |
| Charlson comorbidity index | 0.95 ± 1.51 | 1.14 ± 1.65 ¶ | 1.43 ± 1.82 ¶# | 0.68 ± 1.35 † | | 0.78 ± 1.44 ‡¶ | | 0.96 ± 1.52 §¶# |
| CHA_2_DS_2_VASc score | 2.60 ± 1.55 | 3.09 ± 1.51 ¶ | 3.36 ± 1.47 ¶# | 1.89 ± 1.55 † | | 2.45 ± 1.62 ‡¶ | | 2.71 ± 1.54 §¶# |
| **Concomitant procedures performed during index admission of open-heart cardiac valve surgery** | | | | | | | | |
| CABG | 3396 (37.7%) | 2990 (43.5%) ¶ | 1218 (39.8%) ¶ # | 902 (27.1%) † | | 1111 (32.1%) ‡¶ | | 789 (28.7%) §# |
| PCI | 24 (0.3%) | 37 (0.5%) ¶ | 8 (0.3%) | 11 (0.3%) | | 29 (0.8%) ¶ | | 8 (0.3%) # |
| PPM | 355 (3.9%) | 324 (4.7%) ¶ | 188 (6.1%) ¶ # | 91 (2.7%) † | | 163 (4.7%) ¶ | | 148 (5.4%) ¶ |
| ICD | 29 (0.3%) | 33 (0.5%) | 15 (0.5%) | 36 (1.1%) † | | 47 (1.4%) ‡ | | 33 (1.2%) § |
| Continuous variables are expressed as medians with interquartile range in brackets or mean ± SD; all others represent numbers of patients with values in brackets representing percentages.  AF, atrial fibrillation; ED, Emergency Department; ICU, intensive care unit; IRSAD score, Index of Relative Socioeconomic Advantage and Disadvantage score; ASGSRA, Australian Statistical Geography Standard Remoteness Area; CHF, congestive heart failure; DVT, deep vein thrombosis; PE, pulmonary embolism; CABG, coronary artery bypass graft; PCI, percutaneous coronary intervention; PPM, permanent pacemaker; ICD, implantable cardioverter defibrillator.   - No-AF represents patients without AF; New-AF represents patients with new AF during index admission for cardiac valve surgery and no prior history of AF; Prior-AF represents patients with a history of AF. - P<0.05 comparing MVSx No-AF group to AVSx No-AF group   ‡ P<0.05 comparing MVSx New-AF group to AVSx New-AF group  § P<0.05 comparing MVSx Prior-AF group to AVSx Prior-AF group  ¶ P<0.05 compared to No-AF group (within aortic valve surgery group or separately within mitral valve surgery group)  # P<0.05 compared to New-AF group (within aortic valve surgery group or separately within mitral valve surgery group) | | | | | | | | |

**Supplementary Table S3. Univariable Cox regression analysis of MACE in patients admitted for open-heart cardiac valve surgery**

| **Variables** | **HR** | **95% CI** | **P value** |
| --- | --- | --- | --- |
| Age | 1.06 | 1.05 – 1.06 | < 0.001 |
| Study cohorts: AVSx vs MVSx | 0.76 | 0.73 – 0.79 | < 0.001 |
| AF status |  |  | < 0.001 |
| No AF | Reference |  |  |
| New AF | 1.36 | 1.31 – 1.42 | < 0.001 |
| Prior AF | 2.13 | 2.04 – 2.23 | < 0.001 |
| Males | 0.93 | 0.89 – 0.96 | < 0.001 |
| Year of admission | 0.90 | 0.86 – 0.94 | < 0.001 |
| Referred from ED | 1.50 | 1.41 – 1.58 | < 0.001 |
| ICU admission | 1.07 | 1.01 – 1.12 | 0.02 |
| Hypertension | 1.46 | 1.41 – 1.51 | < 0.001 |
| Hyperlipidaemia | 1.21 | 1.16 – 1.27 | < 0.001 |
| Diabetes | 1.68 | 1.61 – 1.75 | < 0.001 |
| Current/ex-smoker | 1.09 | 1.06 – 1.13 | < 0.001 |
| Congestive heart failure | 2.00 | 1.91 – 2.09 | < 0.001 |
| Pulmonary embolism | 2.08 | 1.59 – 2.73 | < 0.001 |
| Deep venous thrombosis | 1.69 | 1.45 – 1.96 | < 0.001 |
| Ischaemic heart disease | 1.75 | 1.70 – 1.81 | < 0.001 |
| Prior CABG or PCI | 1.80 | 1.70 – 1.91 | < 0.001 |
| Ischaemic stroke | 1.93 | 1.74 – 2.14 | < 0.001 |
| Haemorrhagic stroke | 1.70 | 1.22 – 2.37 | 0.002 |
| Peripheral vascular disease | 1.47 | 1.39 – 1.56 | < 0.001 |
| Malignancy | 1.97 | 1.65 – 2.35 | < 0.001 |
| Sleep apnoea | 1.31 | 1.17 – 1.48 | < 0.001 |
| Chronic kidney disease | 2.52 | 2.38 – 2.67 | < 0.001 |
| Chronic pulmonary disease | 1.86 | 1.74 – 1.98 | < 0.001 |
| Neurodegenerative disease | 1.94 | 1.60 – 2.35 | < 0.001 |
| Major bleed | 1.39 | 1.31 – 1.47 | < 0.001 |
| CABG during admission | 1.66 | 1.61 – 1.72 | < 0.001 |
| PCI during admission | 1.94 | 1.54 – 2.44 | < 0.001 |
| Area of residence based on ASGSRA |  |  |  |
| Metropolitan | Reference |  | < 0.001 |
| Inner regional | 1.08 | 1.04 – 1.12 | < 0.001 |
| Outer regional / remote | 1.16 | 1.08 – 1.25 | < 0.001 |
| IRSAD score | 0.9990 | 0.9987 – 0.9991 | < 0.001 |
| AF, atrial fibrillation; ASGSRA, Australian Statistical Geography Standard Remoteness Area; AVSx, aortic valve surgery; CABG, coronary artery bypass graft; CI, confidence interval; ED, Emergency Department;  HR, hazard ratio; ICU, intensive care unit; IRSAD score, Index of Relative Socioeconomic Advantage and Disadvantage score; MACE, major adverse cardiovascular events defined as all-cause mortality or rehospitalisation with a primary diagnosis of acute myocardial infarction or ischemic cerebrovascular accident or cardiac failure or revascularisation (percutaneous coronary intervention or coronary artery bypass graft), MVSx, mitral valve surgery; PCI, percutaneous coronary intervention. | | | |

**Supplementary Table S4. Univariable Cox regression analysis of all-cause mortality in patients admitted for open-heart cardiac valve surgery**

| **Variables** | **HR** | **95% CI** | **P value** |
| --- | --- | --- | --- |
| Age | 1.07 | 1.07 – 1.08 | < 0.001 |
| Study cohorts: AVSx vs MVSx | 0.69 | 0.66 – 0.72 | < 0.001 |
| AF status |  |  | < 0.001 |
| No AF | Reference |  |  |
| New AF | 1.43 | 1.37 – 1.50 | < 0.001 |
| Prior AF | 2.15 | 2.05 – 2.26 | < 0.001 |
| Males | 0.95 | 0.91 – 0.99 | 0.02 |
| Year of admission | 0.85 | 0.81 – 0.89 | < 0.001 |
| Referred from ED | 1.49 | 1.40 – 1.58 | < 0.001 |
| ICU admission | 1.04 | 0.98 – 1.10 | 0.21 |
| Hypertension | 1.44 | 1.38 – 1.49 | < 0.001 |
| Hyperlipidaemia | 1.21 | 1.15 – 1.28 | < 0.001 |
| Diabetes | 1.65 | 1.58 – 1.73 | < 0.001 |
| Current/ex-smoker | 1.09 | 1.05 – 1.13 | < 0.001 |
| Congestive heart failure | 2.02 | 1.93 – 2.12 | < 0.001 |
| Pulmonary embolism | 2.19 | 1.63 – 2.93 | < 0.001 |
| Deep venous thrombosis | 1.96 | 1.67 – 2.30 | < 0.001 |
| Ischaemic heart disease | 1.81 | 1.74 – 1.88 | < 0.001 |
| Prior CABG or PCI | 1.63 | 1.53 – 1.74 | < 0.001 |
| Ischaemic stroke | 1.95 | 1.74 – 2.19 | < 0.001 |
| Haemorrhagic stroke | 1.61 | 1.10 – 2.34 | 0.01 |
| Peripheral vascular disease | 1.54 | 1.45 – 1.65 | < 0.001 |
| Malignancy | 2.33 | 1.93 – 2.81 | < 0.001 |
| Sleep apnoea | 1.19 | 1.04 – 1.36 | 0.01 |
| Chronic kidney disease | 2.76 | 2.59 – 2.93 | < 0.001 |
| Chronic pulmonary disease | 2.01 | 1.88 – 2.15 | < 0.001 |
| Neurodegenerative disease | 2.45 | 2.01 – 2.98 | < 0.001 |
| Major bleed | 1.46 | 1.37 – 1.56 | < 0.001 |
| CABG | 1.69 | 1.62 – 1.75 | < 0.001 |
| PCI | 1.98 | 1.55 – 2.55 | < 0.001 |
| Area of residence based on ASGSRA |  |  | < 0.001 |
| Metropolitan | Reference |  |  |
| Inner regional | 1.08 | 1.03 – 1.13 | < 0.001 |
| Outer regional / remote | 1.17 | 1.08 – 1.26 | < 0.001 |
| IRSAD score | 0.9990 | 0.9987 – 0.9992 | < 0.001 |
| AF, atrial fibrillation; ASGSRA, Australian Statistical Geography Standard Remoteness Area; AVSx, aortic valve surgery; CABG, coronary artery bypass graft; CI, confidence interval; ED, Emergency Department;  HR, hazard ratio; ICU, intensive care unit; IRSAD score, Index of Relative Socioeconomic Advantage and Disadvantage score; MACE, major adverse cardiovascular events defined as all-cause mortality or rehospitalisation with a primary diagnosis of acute myocardial infarction or ischemic cerebrovascular accident or cardiac failure or revascularisation (percutaneous coronary intervention or coronary artery bypass graft), MVSx, mitral valve surgery; PCI, percutaneous coronary intervention. | | | |

**Supplementary Table S5. Multivariable Cox regression analysis of** **MACE in patients admitted for open-heart cardiac valve surgery: whole study cohort**

| **Variables** | **aHR** | **95% CI** | **P value** |
| --- | --- | --- | --- |
| Age | 1.05 | 1.04 – 1.05 | < 0.001 |
| Study cohorts: AVSx vs MVSx | 0.94 | 0.90 – 0.98 | 0.002 |
| AF status |  |  |  |
| No AF | Reference |  | < 0.001 |
| New AF | 1.11 | 1.07 – 1.16 | < 0.001 |
| Prior AF | 1.68 | 1.60 – 1.76 | < 0.001 |
| Males | 0.94 | 0.90 – 0.97 | < 0.001 |
| Year of admission | 0.88 | 0.84 – 0.92 | < 0.001 |
| Referred from ED | 1.29 | 1.22 – 1.37 | < 0.001 |
| ICU admission | 1.05 | 1.00 – 1.11 | 0.07 |
| Hypertension | 1.05 | 1.01 – 1.09 | 0.007 |
| Hyperlipidaemia | 0.90 | 0.85 – 0.95 | < 0.001 |
| Diabetes | 1.34 | 1.29 – 1.40 | < 0.001 |
| Current/ex-smoker | 1.11 | 1.07 – 1.15 | < 0.001 |
| Congestive heart failure | 1.49 | 1.42 – 1.56 | < 0.001 |
| Pulmonary embolism | 1.39 | 1.05 – 1.83 | 0.02 |
| Deep venous thrombosis | 1.36 | 1.16 – 1.58 | < 0.001 |
| Ischaemic heart disease | 1.11 | 1.06 – 1.17 | < 0.001 |
| Prior CABG or PCI | 1.36 | 1.28 – 1.44 | < 0.001 |
| Ischaemic stroke | 1.67 | 1.50 – 1.86 | < 0.001 |
| Haemorrhagic stroke | 1.31 | 0.93 – 1.85 | 0.12 |
| Peripheral vascular disease | 1.24 | 1.17 – 1.32 | < 0.001 |
| Malignancy | 1.69 | 1.41 – 2.02 | < 0.001 |
| Sleep apnoea | 1.15 | 1.02 – 1.30 | 0.02 |
| Chronic kidney disease | 1.63 | 1.53 – 1.73 | < 0.001 |
| Chronic pulmonary disease | 1.41 | 1.32 – 1.50 | < 0.001 |
| Neurodegenerative disease | 1.29 | 1.06 – 1.56 | 0.01 |
| Major bleed | 1.12 | 1.06 – 1.19 | < 0.001 |
| CABG | 1.13 | 1.08 – 1.18 | < 0.001 |
| PCI | 1.56 | 1.24 – 1.97 | < 0.001 |
| Area of residence based on ASGSRA |  |  | 0.79 |
| Metropolitan | Reference |  |  |
| Inner regional | 1.00 | 0.95 – 1.04 | 0.82 |
| Outer regional / remote | 0.98 | 0.91 – 1.05 | 0.50 |
| IRSAD score | 0.999 | 0.999 – 0.999 | < 0.001 |
| AF, atrial fibrillation; aHR, adjusted hazard ratio; ASGSRA, Australian Statistical Geography Standard Remoteness Area; AVSx, aortic valve surgery; CABG, coronary artery bypass graft; CI, confidence interval; ED, Emergency Department; ICU, intensive care unit; IRSAD score, Index of Relative Socioeconomic Advantage and Disadvantage score; MACE, major adverse cardiovascular events defined as all-cause mortality or rehospitalisation with a primary diagnosis of acute myocardial infarction or ischemic cerebrovascular accident or cardiac failure or revascularisation (percutaneous coronary intervention or coronary artery bypass graft), MVSx, mitral valve surgery; PCI, percutaneous coronary intervention. | | | |

**Supplementary Table S6. Multivariable Cox regression analysis of** **MACE in patients admitted for open-heart aortic valve surgery subgroup**

| **Variables** | **aHR** | **95% CI** | **P value** |
| --- | --- | --- | --- |
| Age | 1.05 | 1.045 – 1.050 | < 0.001 |
| AF status |  |  | < 0.001 |
| No AF | Reference |  |  |
| New AF | 1.14 | 1.08 – 1.19 | < 0.001 |
| Prior AF | 1.76 | 1.67 – 1.86 | < 0.001 |
| Males | 0.99 | 0.94 – 1.03 | 0.54 |
| Year of admission | 0.87 | 0.83 – 0.92 | < 0.001 |
| Referred from ED | 1.28 | 1.19 – 1.37 | < 0.001 |
| ICU admission | 1.08 | 1.01 – 1.15 | 0.02 |
| Hypertension | 1.03 | 0.99 – 1.08 | 0.13 |
| Hyperlipidaemia | 0.88 | 0.83 – 0.93 | < 0.001 |
| Diabetes | 1.32 | 1.26 – 1.38 | < 0.001 |
| Current/ex-smoker | 1.08 | 1.03 – 1.12 | < 0.001 |
| Congestive heart failure | 1.49 | 1.41 – 1.58 | < 0.001 |
| Pulmonary embolism | 1.39 | 0.96 – 2.02 | 0.08 |
| Deep venous thrombosis | 1.39 | 1.15 – 1.67 | < 0.001 |
| Ischaemic heart disease | 1.12 | 1.06 – 1.18 | < 0.001 |
| Prior CABG or PCI | 1.33 | 1.24 – 1.42 | < 0.001 |
| Ischaemic stroke | 1.61 | 1.41 – 1.84 | < 0.001 |
| Haemorrhagic stroke | 1.94 | 1.24 – 3.05 | 0.004 |
| Peripheral vascular disease | 1.17 | 1.09 – 1.25 | < 0.001 |
| Malignancy | 1.75 | 1.42 – 2.16 | < 0.001 |
| Sleep apnoea | 1.22 | 1.06 – 1.40 | 0.005 |
| Chronic kidney disease | 1.61 | 1.50 – 1.73 | < 0.001 |
| Chronic pulmonary disease | 1.34 | 1.24 – 1.45 | < 0.001 |
| Neurodegenerative disease | 1.36 | 1.10 – 1.67 | 0.004 |
| Major bleed | 1.11 | 1.04 – 1.20 | 0.003 |
| CABG | 1.06 | 1.00 – 1.12 | 0.04 |
| PCI | 1.27 | 0.92 – 1.74 | 0.14 |
| Area of residence based on ASGSRA |  |  | 0.91 |
| Metropolitan | Reference |  |  |
| Inner regional | 1.01 | 0.96 – 1.06 | 0.71 |
| Outer regional / remote | 0.99 | 0.91 – 1.09 | 0.90 |
| IRSAD score | 0.999 | 0.999 – 1.000 | < 0.001 |
| AF, atrial fibrillation; aHR, adjusted hazard ratio; ASGSRA, Australian Statistical Geography Standard Remoteness Area; AVSx, aortic valve surgery; CABG, coronary artery bypass graft; CI, confidence interval; ED, Emergency Department; ICU, intensive care unit; IRSAD score, Index of Relative Socioeconomic Advantage and Disadvantage score; MACE, major adverse cardiovascular events defined as all-cause mortality or rehospitalisation with a primary diagnosis of acute myocardial infarction or ischemic cerebrovascular accident or cardiac failure or revascularisation (percutaneous coronary intervention or coronary artery bypass graft), MVSx, mitral valve surgery; PCI, percutaneous coronary intervention. | | | |

**Supplementary Table S7. Multivariable Cox regression analysis of MACE in patients admitted for open-heart mitral valve surgery subgroup**

| **Variables** | **aHR** | **95% CI** | **P value** |
| --- | --- | --- | --- |
| Age | 1.05 | 1.04 –1.05 | < 0.001 |
| AF status |  |  |  |
| No AF | Reference |  | < 0.001 |
| New AF | 1.05 | 0.97 – 1.14 | 0.27 |
| Prior AF | 1.51 | 1.39 – 1.64 | < 0.001 |
| Males | 0.85 | 0.79 – 0.91 | < 0.001 |
| Year of admission | 0.88 | 0.82 – 0.95 | 0.001 |
| Referred from ED | 1.29 | 1.17 – 1.43 | < 0.001 |
| ICU admission | 0.99 | 0.90 – 1.09 | 0.89 |
| Hypertension | 1.08 | 1.01 – 1.16 | 0.02 |
| Hyperlipidaemia | 0.93 | 0.83 – 1.03 | 0.15 |
| Diabetes | 1.44 | 1.32 – 1.57 | < 0.001 |
| Current/ex-smoker | 1.16 | 1.08 – 1.23 | < 0.001 |
| Congestive heart failure | 1.47 | 1.36 – 1.59 | < 0.001 |
| Pulmonary embolism | 1.52 | 1.00 – 2.31 | 0.048 |
| Deep venous thrombosis | 1.28 | 0.98 – 1.67 | 0.07 |
| Ischaemic heart disease | 1.11 | 1.01 – 1.21 | 0.02 |
| Prior CABG or PCI | 1.42 | 1.25 – 1.61 | < 0.001 |
| Ischaemic stroke | 1.72 | 1.43 – 2.07 | < 0.001 |
| Haemorrhagic stroke | 0.96 | 0.55 – 1.66 | 0.88 |
| Peripheral vascular disease | 1.51 | 1.33 – 1.71 | < 0.001 |
| Malignancy | 1.60 | 1.14 – 2.25 | 0.006 |
| Sleep apnoea | 0.97 | 0.76 – 1.25 | 0.84 |
| Chronic kidney disease | 1.68 | 1.49 – 1.88 | < 0.001 |
| Chronic pulmonary disease | 1.57 | 1.39 – 1.78 | < 0.001 |
| Neurodegenerative disease | 1.03 | 0.62 – 1.71 | 0.91 |
| Major bleed | 1.13 | 1.01 – 1.26 | 0.03 |
| CABG | 1.28 | 1.17 – 1.39 | < 0.001 |
| PCI | 1.97 | 1.39 – 2.79 | < 0.001 |
| Area of residence based on ASGSRA |  |  | 0.63 |
| Metropolitan | Reference |  |  |
| Inner regional | 0.97 | 0.90 – 1.05 | 0.48 |
| Outer regional / remote | 0.95 | 0.83 – 1.09 | 0.44 |
| IRSAD score | 0.999 | 0.998 – 0.999 | < 0.001 |
| AF, atrial fibrillation; aHR, adjusted hazard ratio; ASGSRA, Australian Statistical Geography Standard Remoteness Area; AVSx, aortic valve surgery; CABG, coronary artery bypass graft; CI, confidence interval; ED, Emergency Department; ICU, intensive care unit; IRSAD score, Index of Relative Socioeconomic Advantage and Disadvantage score; MACE, major adverse cardiovascular events defined as all-cause mortality or rehospitalisation with a primary diagnosis of acute myocardial infarction or ischemic cerebrovascular accident or cardiac failure or revascularisation (percutaneous coronary intervention or coronary artery bypass graft), MVSx, mitral valve surgery; PCI, percutaneous coronary intervention. | | | |

**Supplementary Table S8. Multivariable Cox regression analysis of all-cause mortality in patients admitted for open-heart cardiac valve surgery: whole study cohort**

| **Variables** | **aHR** | **95% CI** | **P value** |
| --- | --- | --- | --- |
| Age | 1.07 | 1.06 – 1.07 | < 0.001 |
| Study cohorts: AVSx vs MVSx | 0.89 | 0.85 – 0.94 | < 0.001 |
| AF status |  |  | < 0.001 |
| No AF | Reference |  |  |
| New AF | 1.13 | 1.08 – 1.18 | < 0.001 |
| Prior AF | 1.62 | 1.54 – 1.70 | < 0.001 |
| Males | 1.01 | 0.96 – 1.05 | 0.79 |
| Year of Admission | 0.81 | 0.77 – 0.85 | < 0.001 |
| Referred from ED | 1.28 | 1.20 – 1.36 | < 0.001 |
| Hypertension | 0.98 | 0.94 – 1.02 | 0.33 |
| Hyperlipidaemia | 0.90 | 0.85 – 0.95 | < 0.001 |
| Diabetes | 1.36 | 1.30 – 1.43 | < 0.001 |
| Current/ex-smoker | 1.12 | 1.08 – 1.17 | < 0.001 |
| Congestive heart failure | 1.49 | 1.42 – 1.57 | < 0.001 |
| Pulmonary embolism | 1.56 | 1.15 – 2.10 | 0.004 |
| Deep venous thrombosis | 1.60 | 1.36 – 1.88 | < 0.001 |
| Ischaemic heart disease | 1.13 | 1.07 – 1.19 | < 0.001 |
| Prior CABG or PCI | 1.18 | 1.10 – 1.26 | < 0.001 |
| Ischaemic stroke | 1.70 | 1.51 – 1.92 | < 0.001 |
| Haemorrhagic stroke | 1.12 | 0.76 – 1.66 | 0.56 |
| Peripheral vascular disease | 1.28 | 1.19 – 1.36 | < 0.001 |
| Malignancy | 2.08 | 1.72 – 2.51 | < 0.001 |
| Sleep apnoea | 1.09 | 0.95 – 1.25 | 0.23 |
| Chronic kidney disease | 1.71 | 1.60 – 1.82 | < 0.001 |
| Chronic pulmonary disease | 1.52 | 1.42 – 1.63 | < 0.001 |
| Neurodegenerative disease | 1.64 | 1.34 – 1.99 | < 0.001 |
| Major bleed | 1.15 | 1.08 – 1.23 | < 0.001 |
| CABG | 1.08 | 1.03 – 1.14 | 0.002 |
| PCI | 1.57 | 1.22 – 2.02 | < 0.001 |
| Area of residence based on ASGSRA |  |  | 0.65 |
| Metropolitan | Reference |  |  |
| Inner regional | 0.99 | 0.94 – 1.04 | 0.64 |
| Outer regional / remote | 0.96 | 0.89 – 1.05 | 0.37 |
| IRSAD score | 0.998 | 0.998 – 0.999 | < 0.001 |
| AF, atrial fibrillation; aHR, adjusted hazard ratio; ASGSRA, Australian Statistical Geography Standard Remoteness Area; AVSx, aortic valve surgery; CABG, coronary artery bypass graft; CI, confidence interval; ED, Emergency Department; ICU, intensive care unit; IRSAD score, Index of Relative Socioeconomic Advantage and Disadvantage score; MACE, major adverse cardiovascular events defined as all-cause mortality or rehospitalisation with a primary diagnosis of acute myocardial infarction or ischemic cerebrovascular accident or cardiac failure or revascularisation (percutaneous coronary intervention or coronary artery bypass graft), MVSx, mitral valve surgery; PCI, percutaneous coronary intervention. | | | |

**Supplementary Table S9. Multivariable Cox regression analysis of all-cause mortality in patients admitted for open-heart aortic valve surgery subgroup**

| **Variables** | **aHR** | **95% CI** | **P value** |
| --- | --- | --- | --- |
| Age | 1.07 | 1.06 – 1.07 | < 0.001 |
| AF status |  |  | < 0.001 |
| No AF | Reference |  |  |
| New AF | 1.16 | 1.10 – 1.22 | < 0.001 |
| Prior AF | 1.69 | 1.59 – 1.79 | < 0.001 |
| Males | 1.05 | 1.00 – 1.10 | 0.061 |
| Year of admission | 0.81 | 0.77 – 0.86 | < 0.001 |
| Referred from ED | 1.27 | 1.17 – 1.37 | < 0.001 |
| Hypertension | 0.97 | 0.92 – 1.01 | 0.17 |
| Hyperlipidaemia | 0.89 | 0.84 – 0.95 | < 0.001 |
| Diabetes | 1.34 | 1.27 – 1.42 | < 0.001 |
| Current/ex-smoker | 1.08 | 1.03 – 1.13 | 0.001 |
| Congestive heart failure | 1.48 | 1.39 – 1.58 | < 0.001 |
| Pulmonary embolism | 1.56 | 1.05 – 2.32 | 0.03 |
| Deep venous thrombosis | 1.70 | 1.40 – 2.07 | < 0.001 |
| Ischaemic heart disease | 1.12 | 1.06 – 1.19 | < 0.001 |
| Prior CABG or PCI | 1.15 | 1.06 – 1.24 | < 0.001 |
| Ischaemic stroke | 1.66 | 1.44 – 1.92 | < 0.001 |
| Haemorrhagic stroke | 1.19 | 0.71 – 2.00 | 0.50 |
| Peripheral vascular disease | 1.17 | 1.08 – 1.26 | < 0.001 |
| Malignancy | 2.27 | 1.82 – 2.82 | < 0.001 |
| Sleep apnoea | 1.12 | 0.96 – 1.32 | 0.15 |
| Chronic kidney disease | 1.67 | 1.55 – 1.80 | < 0.001 |
| Chronic pulmonary disease | 1.42 | 1.31 – 1.54 | < 0.001 |
| Neurodegenerative disease | 1.72 | 1.39 – 2.13 | < 0.001 |
| Major bleed | 1.14 | 1.05 – 1.23 | 0.001 |
| CABG | 1.02 | 0.96 – 1.08 | 0.54 |
| PCI | 1.38 | 0.99 – 1.94 | 0.06 |
| Area of residence based on ASGSRA |  |  | 0.78 |
| Metropolitan | Reference |  |  |
| Inner regional | 1.02 | 0.96 – 1.08 | 0.54 |
| Outer regional / remote | 0.99 | 0.90 – 1.09 | 0.86 |
| IRSAD score | 0.999 | 0.999 – 1.000 | < 0.001 |
| AF, atrial fibrillation; aHR, adjusted hazard ratio; ASGSRA, Australian Statistical Geography Standard Remoteness Area; AVSx, aortic valve surgery; CABG, coronary artery bypass graft; CI, confidence interval; ED, Emergency Department; ICU, intensive care unit; IRSAD score, Index of Relative Socioeconomic Advantage and Disadvantage score; MACE, major adverse cardiovascular events defined as all-cause mortality or rehospitalisation with a primary diagnosis of acute myocardial infarction or ischemic cerebrovascular accident or cardiac failure or revascularisation (percutaneous coronary intervention or coronary artery bypass graft), MVSx, mitral valve surgery; PCI, percutaneous coronary intervention. | | | |

**Supplementary Table S10. Multivariable Cox regression analysis of all-cause mortality in patients admitted for open-heart mitral valve surgery subgroup**

| **Variables** | **aHR** | **95% CI** | **P value** |
| --- | --- | --- | --- |
| Age | 1.06 | 1.06 – 1.07 | < 0.001 |
| AF status |  |  | < 0.001 |
| No AF | Reference |  |  |
| New AF | 1.06 | 0.96 – 1.17 | 0.24 |
| Prior AF | 1.47 | 1.33 – 1.61 | < 0.001 |
| Males | 0.92 | 0.85 – 0.99 | 0.035 |
| Year of Admission | 0.78 | 0.71 – 0.86 | < 0.001 |
| Referred from ED | 1.27 | 1.13 – 1.43 | < 0.001 |
| Hypertension | 0.998 | 0.92 – 1.08 | 0.95 |
| Hyperlipidaemia | 0.91 | 0.81 – 1.02 | 0.11 |
| Diabetes | 1.43 | 1.29 – 1.58 | < 0.001 |
| Current and ex-smoker | 1.20 | 1.11 – 1.29 | < 0.001 |
| Congestive heart failure | 1.51 | 1.38 – 1.64 | < 0.001 |
| Pulmonary embolism | 1.58 | 1.00 – 2.50 | 0.050 |
| Deep venous thrombosis | 1.34 | 0.99 – 1.80 | 0.055 |
| Ischaemic heart disease | 1.14 | 1.04 – 1.26 | 0.008 |
| Prior CABG or PCI | 1.23 | 1.06 – 1.42 | 0.006 |
| Ischaemic stroke | 1.75 | 1.42 – 2.16 | < 0.001 |
| Haemorrhagic stroke | 1.16 | 0.62 – 2.16 | 0.64 |
| Peripheral vascular disease | 1.79 | 1.56 – 2.05 | < 0.001 |
| Malignancy | 1.70 | 1.16 – 2.49 | 0.006 |
| Sleep apnoea | 0.996 | 0.75 – 1.32 | 0.98 |
| Chronic kidney disease | 1.87 | 1.65 – 2.13 | < 0.001 |
| Chronic pulmonary disease | 1.81 | 1.59 – 2.06 | < 0.001 |
| Neurodegenerative disease | 1.40 | 0.84 – 2.32 | 0.20 |
| Major bleed | 1.19 | 1.05 – 1.34 | 0.006 |
| CABG | 1.22 | 1.10 – 1.34 | < 0.001 |
| PCI | 1.97 | 1.33 – 2.92 | < 0.001 |
| Area of residence based on ASGSRA |  |  | 0.18 |
| Metropolitan | Reference |  |  |
| Inner regional | 0.92 | 0.84 – 1.01 | 0.08 |
| Outer regional / remote | 0.93 | 0.79 – 1.09 | 0.35 |
| IRSAD score | 0.998 | 0.998 – 0.999 | < 0.001 |
| AF, atrial fibrillation; aHR, adjusted hazard ratio; ASGSRA, Australian Statistical Geography Standard Remoteness Area; AVSx, aortic valve surgery; CABG, coronary artery bypass graft; CI, confidence interval; ED, Emergency Department; ICU, intensive care unit; IRSAD score, Index of Relative Socioeconomic Advantage and Disadvantage score; MACE, major adverse cardiovascular events defined as all-cause mortality or rehospitalisation with a primary diagnosis of acute myocardial infarction or ischemic cerebrovascular accident or cardiac failure or revascularisation (percutaneous coronary intervention or coronary artery bypass graft), MVSx, mitral valve surgery; PCI, percutaneous coronary intervention. | | | |

**Supplementary Table S11. Multivariable Cox regression analysis of MACE in patients admitted for open-heart aortic valve surgery subgroup (with AVSx subtypes)**

| **Variables** | **aHR** | **95% CI** | **P value** |
| --- | --- | --- | --- |
| Age | 1.05 | 1.045 – 1.050 | < 0.001 |
| AVSx categories: Bioprosthetic vs mechanical | 1.003 | 0.95 – 1.06 | 0.93 |
| AF status |  |  |  |
| No AF | Reference |  | < 0.001 |
| New AF | 1.13 | 1.08 – 1.19 | < 0.001 |
| Prior AF | 1.76 | 1.67 – 1.86 | < 0.001 |
| Males | 0.99 | 0.95 – 1.04 | 0.64 |
| Year of admission | 0.87 | 0.82 – 0.91 | < 0.001 |
| Referred from ED | 1.27 | 1.18 – 1.36 | < 0.001 |
| ICU admission | 1.10 | 1.03 – 1.18 | 0.005 |
| Hypertension | 1.04 | 0.99 – 1.08 | 0.12 |
| Hyperlipidaemia | 0.88 | 0.83 – 0.93 | < 0.001 |
| Diabetes | 1.32 | 1.26 – 1.38 | < 0.001 |
| Current/ex-smoker | 1.08 | 1.04 – 1.13 | < 0.001 |
| Congestive heart failure | 1.49 | 1.41 – 1.58 | < 0.001 |
| Pulmonary embolism | 1.30 | 0.88 – 1.92 | 0.19 |
| Deep venous thrombosis | 1.40 | 1.16 – 1.69 | < 0.001 |
| Ischaemic heart disease | 1.12 | 1.06 – 1.18 | < 0.001 |
| Prior CABG or PCI | 1.33 | 1.24 – 1.42 | < 0.001 |
| Ischaemic stroke | 1.64 | 1.44 – 1.88 | < 0.001 |
| Haemorrhagic stroke | 1.95 | 1.23 – 3.09 | 0.005 |
| Peripheral vascular disease | 1.18 | 1.10 – 1.26 | < 0.001 |
| Malignancy | 1.66 | 1.33 – 2.06 | < 0.001 |
| Sleep apnoea | 1.22 | 1.06 – 1.40 | 0.005 |
| Chronic kidney disease | 1.61 | 1.50 – 1.73 | < 0.001 |
| Chronic pulmonary disease | 1.34 | 1.24 – 1.45 | < 0.001 |
| Neurodegenerative disease | 1.35 | 1.09 – 1.66 | 0.005 |
| Major bleed | 1.10 | 1.03 – 1.19 | 0.008 |
| CABG during admission | 1.06 | 1.003 – 1.119 | 0.040 |
| PCI during admission | 1.22 | 0.88 – 1.70 | 0.243 |
| Area of residence based on ASGSRA |  |  |  |
| Metropolitan | Reference |  | 0.921 |
| Inner regional | 1.01 | 0.96 – 1.06 | 0.76 |
| Outer regional / remote | 0.99 | 0.91 – 1.08 | 0.85 |
| IRSAD score | 0.999 | 0.9999 – 1.000 | < 0.01 |
| AF, atrial fibrillation; aHR, adjusted hazard ratio; ASGSRA, Australian Statistical Geography Standard Remoteness Area; AVSx, aortic valve surgery; CABG, coronary artery bypass graft; CI, confidence interval; ED, Emergency Department; ICU, intensive care unit; IRSAD score, Index of Relative Socioeconomic Advantage and Disadvantage score; MACE, major adverse cardiovascular events defined as all-cause mortality or rehospitalisation with a primary diagnosis of acute myocardial infarction or ischemic cerebrovascular accident or cardiac failure or revascularisation (percutaneous coronary intervention or coronary artery bypass graft); PCI, percutaneous coronary intervention. | | | |

**Supplementary Table S12. Multivariable Cox regression analysis of MACE in patients admitted for open-heart mitral valve surgery subgroup (with MVSx subtypes)**

| **Variables** | **aHR** | **95% CI** | **P value** |
| --- | --- | --- | --- |
| Age | 1.04 | 1.04 – 1.05 | < 0.001 |
| MVSx categories |  |  |  |
| Repair | Reference |  | < 0.001 |
| Bioprosthetic | 1.56 | 1.41 – 1.72 | < 0.001 |
| Mechanical | 1.42 | 1.27 – 1.58 | < 0.001 |
| AF status |  |  |  |
| No AF | Reference |  | < 0.001 |
| New AF | 1.04 | 0.94 – 1.14 | 0.50 |
| Prior AF | 1.41 | 1.27 – 1.56 | < 0.001 |
| Males | 0.88 | 0.82 – 0.96 | 0.002 |
| Year of admission | 0.90 | 0.82 – 0.99 | 0.037 |
| Referred from ED | 1.21 | 1.07 – 1.38 | 0.003 |
| ICU admission | 1.01 | 0.91 – 1.13 | 0.79 |
| Hypertension | 1.10 | 1.01 – 1.19 | 0.028 |
| Hyperlipidaemia | 0.97 | 0.85 – 1.11 | 0.69 |
| Diabetes | 1.40 | 1.26 – 1.56 | < 0.001 |
| Current/ex-smoker | 1.14 | 1.05 – 1.23 | 0.002 |
| Congestive heart failure | 1.42 | 1.30 – 1.56 | < 0.001 |
| Pulmonary embolism | 1.71 | 1.06 – 2.75 | 0.028 |
| Deep venous thrombosis | 1.28 | 0.94 – 1.74 | 0.12 |
| Ischaemic heart disease | 1.08 | 0.97 – 1.20 | 0.17 |
| Prior CABG or PCI | 1.40 | 1.19 – 1.64 | < 0.001 |
| Ischaemic stroke | 1.84 | 1.48 – 2.29 | < 0.001 |
| Haemorrhagic stroke | 0.79 | 0.42 – 1.50 | 0.48 |
| Peripheral vascular disease | 1.37 | 1.18 – 1.60 | < 0.001 |
| Malignancy | 1.94 | 1.32 – 2.84 | < 0.001 |
| Sleep apnoea | 1.12 | 0.84 - 1.51 | 0.44 |
| Chronic kidney disease | 1.63 | 1.41 – 1.88 | < 0.001 |
| Chronic pulmonary disease | 1.67 | 1.44 – 1.94 | < 0.001 |
| Neurodegenerative disease | 1.09 | 0.64 – 1.84 | 0.76 |
| Major bleed | 1.11 | 0.97 – 1.26 | 0.13 |
| CABG during admission | 1.21 | 1.08 – 1.35 | < 0.001 |
| PCI during admission | 1.96 | 1.26 – 3.04 | 0.003 |
| Area of residence based on ASGSRA |  |  |  |
| Metropolitan | Reference |  | 0.92 |
| Inner regional | 0.98 | 0.89 – 1.08 | 0.68 |
| Outer regional / remote | 0.99 | 0.84 – 1.17 | 0.92 |
| IRSAD score | 0.999 | 0.998 – 0.999 | < 0.001 |
| AF, atrial fibrillation; aHR, adjusted hazard ratio; ASGSRA, Australian Statistical Geography Standard Remoteness Area; CABG, coronary artery bypass graft; CI, confidence interval; ED, Emergency Department; ICU, intensive care unit; IRSAD score, Index of Relative Socioeconomic Advantage and Disadvantage score; MACE, major adverse cardiovascular events defined as all-cause mortality or rehospitalisation with a primary diagnosis of acute myocardial infarction or ischemic cerebrovascular accident or cardiac failure or revascularisation (percutaneous coronary intervention or coronary artery bypass graft); MVSx, mitral valve surgery; PCI, percutaneous coronary intervention. | | | |

**Supplementary Table S13. Multivariable Cox regression analysis of all-cause mortality in patients admitted for open-heart aortic valve surgery subgroup (with AVSx subtypes)**

| **Variables** | **aHR** | **95% CI** | **P value** |
| --- | --- | --- | --- |
| Age | 1.07 | 1.06 – 1.07 | < 0.001 |
| AVSx categories: Bioprosthetic vs mechanical | 1.00 | 0.94 – 1.07 | 0.91 |
| AF status |  |  |  |
| No AF | Reference |  | < 0.001 |
| New AF | 1.15 | 1.09 – 1.21 | < 0.001 |
| Prior AF | 1.68 | 1.58 – 1.79 | < 0.001 |
| Males | 1.05 | 1.002 – 1.11 | 0.043 |
| Year of admission | 0.81 | 0.77 – 0.87 | < 0.001 |
| Referred from ED | 1.26 | 1.17 – 1.37 | < 0.001 |
| Hypertension | 0.97 | 0.92 – 1.02 | 0.19 |
| Hyperlipidaemia | 0.89 | 0.83 – 0.95 | < 0.001 |
| Diabetes | 1.35 | 1.28 – 1.42 | < 0.001 |
| Current/ex-smoker | 1.08 | 1.03 – 1.14 | 0.001 |
| Congestive heart failure | 1.48 | 1.39 – 1.57 | < 0.001 |
| Pulmonary embolism | 1.58 | 1.06 – 2.38 | 0.027 |
| Deep venous thrombosis | 1.69 | 1.38 – 2.06 | < 0.001 |
| Ischaemic heart disease | 1.13 | 1.06 – 1.20 | < 0.001 |
| Prior CABG or PCI | 1.14 | 1.05 – 1.23 | 0.001 |
| Ischaemic stroke | 1.69 | 1.46 – 1.96 | < 0.001 |
| Haemorrhagic stroke | 1.15 | 0.68 – 1.96 | 0.60 |
| Peripheral vascular disease | 1.18 | 1.09 – 1.27 | < 0.001 |
| Malignancy | 2.19 | 1.75 – 2.74 | < 0.001 |
| Sleep apnoea | 1.13 | 0.96 – 1.32 | 0.14 |
| Chronic kidney disease | 1.66 | 1.54 – 1.79 | < 0.001 |
| Chronic pulmonary disease | 1.43 | 1.31 – 1.55 | < 0.001 |
| Neurodegenerative disease | 1.71 | 1.38 – 2.13 | < 0.001 |
| Major bleed | 1.12 | 1.04 – 1.21 | 0.005 |
| CABG during admission | 1.02 | 0.96 – 1.08 | 0.63 |
| PCI during admission | 1.32 | 0.93 – 1.87 | 0.13 |
| Area of residence based on ASGSRA |  |  |  |
| Metropolitan | Reference |  | 0.80 |
| Inner regional | 1.02 | 0.96 – 1.08 | 0.55 |
| Outer regional / remote | 1.00 | 0.90 – 1.10 | 0.92 |
| IRSAD score | 0.999 | 0.999 – 1.000 | < 0.001 |
| AF, atrial fibrillation; aHR, adjusted hazard ratio; ASGSRA, Australian Statistical Geography Standard Remoteness Area; AVSx, aortic valve surgery; CABG, coronary artery bypass graft; CI, confidence interval; ED, Emergency Department; ICU, intensive care unit; IRSAD score, Index of Relative Socioeconomic Advantage and Disadvantage score; MACE, major adverse cardiovascular events defined as all-cause mortality or rehospitalisation with a primary diagnosis of acute myocardial infarction or ischemic cerebrovascular accident or cardiac failure or revascularisation (percutaneous coronary intervention or coronary artery bypass graft); PCI, percutaneous coronary intervention. | | | |

**Supplementary Table S14. Multivariable Cox regression analysis of all-cause mortality in patients admitted for open-heart mitral valve surgery subgroup (with MVSx subtypes)**

| **Variables** | **aHR** | **95% CI** | **P value** |
| --- | --- | --- | --- |
| Age | 1.06 | 1.06 – 1.07 | < 0.001 |
| MVSx categories |  |  |  |
| Repair | Reference |  | < 0.001 |
| Bioprosthetic | 1.64 | 1.46 – 1.83 | < 0.001 |
| Mechanical | 1.44 | 1.26 – 1.63 | < 0.001 |
| AF status |  |  |  |
| No AF | Reference |  | < 0.001 |
| New AF | 1.04 | 0.93 – 1.17 | 0.50 |
| Prior AF | 1.38 | 1.23 – 1.55 | < 0.001 |
| Males | 0.96 | 0.88 – 1.06 | 0.42 |
| Year of Admission | 0.77 | 0.69 – 0.87 | < 0.001 |
| Referred from ED | 1.26 | 1.09 – 1.46 | 0.002 |
| Hypertension | 1.03 | 0.94 – 1.13 | 0.57 |
| Hyperlipidaemia | 0.95 | 0.82 – 1.10 | 0.48 |
| Diabetes | 1.37 | 1.21 – 1.56 | < 0.001 |
| Current and ex-smoker | 1.17 | 1.06 – 1.28 | 0.001 |
| Congestive heart failure | 1.43 | 1.29 – 1.59 | < 0.001 |
| Pulmonary embolism | 2.20 | 1.30 – 3.73 | 0.003 |
| Deep venous thrombosis | 1.37 | 0.97 – 1.94 | 0.075 |
| Ischaemic heart disease | 1.09 | 0.96 – 1.23 | 0.18 |
| Prior CABG or PCI | 1.33 | 1.12 – 1.59 | 0.002 |
| Ischaemic stroke | 1.95 | 1.52 – 2.50 | < 0.001 |
| Haemorrhagic stroke | 0.73 | 0.34 – 1.54 | 0.41 |
| Peripheral vascular disease | 1.60 | 1.35 – 1.90 | < 0.001 |
| Malignancy | 2.18 | 1.43 – 3.33 | < 0.001 |
| Sleep apnoea | 1.15 | 0.83 – 1.61 | 0.40 |
| Chronic kidney disease | 1.81 | 1.54 – 2.12 | < 0.001 |
| Chronic pulmonary disease | 1.86 | 1.58 – 2.18 | < 0.001 |
| Neurodegenerative disease | 1.42 | 0.84 – 2.41 | 0.20 |
| Major bleed | 1.14 | 0.99 – 1.31 | 0.077 |
| CABG during admission | 1.22 | 1.08 – 1.38 | 0.001 |
| PCI during admission | 1.87 | 1.15 – 3.06 | 0.012 |
| Area of residence based on ASGSRA |  |  |  |
| Metropolitan | Reference |  | 0.63 |
| Inner regional | 0.95 | 0.84 – 1.06 | 0.34 |
| Outer regional / remote | 0.97 | 0.80 – 1.17 | 0.72 |
| IRSAD score | 0.999 | 0.998 – 0.999 | < 0.001 |
| AF, atrial fibrillation; aHR, adjusted hazard ratio; ASGSRA, Australian Statistical Geography Standard Remoteness Area; CABG, coronary artery bypass graft; CI, confidence interval; ED, Emergency Department; ICU, intensive care unit; IRSAD score, Index of Relative Socioeconomic Advantage and Disadvantage score; MACE, major adverse cardiovascular events defined as all-cause mortality or rehospitalisation with a primary diagnosis of acute myocardial infarction or ischemic cerebrovascular accident or cardiac failure or revascularisation (percutaneous coronary intervention or coronary artery bypass graft); MVSx, mitral valve surgery; PCI, percutaneous coronary intervention. | | | |

**Supplementary Table S15. Interaction term univariable Cox regression analysis of MACE in patients admitted for open-heart cardiac valve surgery**

| **Variables** | **HR** | **95% CI** | **P value** |
| --- | --- | --- | --- |
| AF status (No AF)*Type of cardiac valve surgery | Reference |  | < 0.001 |
| AF status (New AF)*Type of cardiac valve surgery | 0.81 | 0.76 – 0.85 | < 0.001 |
| AF status (Prior AF)*Type of cardiac valve surgery | 1.27 | 1.20 – 1.34 | < 0.001 |
| AF, atrial fibrillation; HR, hazard ratio; CI, confidence interval | | | |

**Supplementary Table S16. Interaction term multivariable Cox regression analysis of MACE in patients admitted for open-heart cardiac valve surgery**

| **Variables** | **aHR** | **95% CI** | **P value** |
| --- | --- | --- | --- |
| Age | 1.05 | 1.04 – 1.05 | < 0.001 |
| Study cohorts: AVSx vs MVSx | 0.99 | 0.93 – 1.07 | 0.85 |
| AF status |  |  |  |
| No AF | Reference |  | < 0.001 |
| New AF | 1.13 | 1.08 – 1.18 | < 0.001 |
| Prior AF | 1.73 | 1.64 – 1.83 | < 0.001 |
| Males | 0.94 | 0.90 – 0.97 | < 0.001 |
| Year of admission | 0.88 | 0.84 – 0.92 | < 0.001 |
| Referred from ED | 1.29 | 1.22 – 1.37 | < 0.001 |
| ICU admission | 1.05 | 0.995 – 1.11 | 0.074 |
| Hypertension | 1.05 | 1.01 – 1.09 | 0.008 |
| Hyperlipidaemia | 0.90 | 0.85 – 0.95 | < 0.001 |
| Diabetes | 1.34 | 1.29 – 1.40 | < 0.001 |
| Current/ex-smoker | 1.11 | 1.07 – 1.15 | < 0.001 |
| Congestive heart failure | 1.49 | 1.42 – 1.56 | < 0.001 |
| Pulmonary embolism | 1.39 | 1.06 – 1.84 | 0.018 |
| Deep venous thrombosis | 1.35 | 1.16 – 1.58 | < 0.001 |
| Ischaemic heart disease | 1.11 | 1.06 – 1.17 | < 0.001 |
| Prior CABG or PCI | 1.36 | 1.28 – 1.44 | < 0.001 |
| Ischaemic stroke | 1.67 | 1.50 – 1.86 | < 0.001 |
| Haemorrhagic stroke | 1.31 | 0.93 – 1.84 | 0.127 |
| Peripheral vascular disease | 1.24 | 1.17 – 1.32 | < 0.001 |
| Malignancy | 1.69 | 1.42 – 2.02 | < 0.001 |
| Sleep apnoea | 1.15 | 1.02 – 1.30 | 0.02 |
| Chronic kidney disease | 1.63 | 1.53 – 1.72 | < 0.001 |
| Chronic pulmonary disease | 1.41 | 1.32 – 1.50 | < 0.001 |
| Neurodegenerative disease | 1.29 | 1.06 – 1.56 | 0.011 |
| Major bleed | 1.12 | 1.06 – 1.19 | < 0.001 |
| CABG | 1.13 | 1.08 – 1.18 | < 0.001 |
| PCI | 1.56 | 1.24 – 1.97 | < 0.001 |
| Area of residence based on ASGSRA |  |  |  |
| Metropolitan | Reference |  | 0.799 |
| Inner regional | 0.995 | 0.95 – 1.04 | 0.83 |
| Outer regional / remote | 0.98 | 0.91 – 1.05 | 0.51 |
| IRSAD score | 0.999 | 0.999 – 0.999 | < 0.001 |
| AF status (No AF)*Type of cardiac valve surgery | Reference |  | 0.093 |
| AF status (New AF)*Type of cardiac valve surgery | 0.94 | 0.86 – 1.03 | 0.198 |
| AF status (Prior AF)*Type of cardiac valve surgery | 0.90 | 0.82 – 0.99 | 0.03 |
| AF, atrial fibrillation; aHR, adjusted hazard ratio; ASGSRA, Australian Statistical Geography Standard Remoteness Area; AVSx, aortic valve surgery; CABG, coronary artery bypass graft; CI, confidence interval; ED, Emergency Department; ICU, intensive care unit; IRSAD score, Index of Relative Socioeconomic Advantage and Disadvantage score; MACE, major adverse cardiovascular events defined as all-cause mortality or rehospitalisation with a primary diagnosis of acute myocardial infarction or ischemic cerebrovascular accident or congestive heart failure or revascularisation (percutaneous coronary intervention or coronary artery bypass graft), MVSx, mitral valve surgery; PCI, percutaneous coronary intervention. | | | |

**Supplementary Table S17. Interaction term univariable Cox regression analysis of all-cause mortality in patients admitted for open-heart cardiac valve surgery**

| **Variables** | **HR** | **95% CI** | **P value** |
| --- | --- | --- | --- |
| AF status (No AF)*Type of cardiac valve surgery | Reference |  | < 0.001 |
| AF status (New AF)*Type of cardiac valve surgery | 0.76 | 0.71 – 0.81 | < 0.001 |
| AF status (Prior AF)*Type of cardiac valve surgery | 1.15 | 1.08 – 1.22 | < 0.001 |
| AF, atrial fibrillation; HR, hazard ratio; CI, confidence interval | | | |

**Supplementary Table S18. Interaction term multivariable Cox regression analysis of all-cause mortality in patients admitted for open-heart cardiac valve surgery**

| **Variables** | **aHR** | **95% CI** | **P value** |
| --- | --- | --- | --- |
| Age | 1.07 | 1.06 – 1.07 | < 0.001 |
| Study cohorts: AVSx vs MVSx | 0.95 | 0.88 – 1.03 | 0.243 |
| AF status |  |  | < 0.001 |
| No AF | Reference |  | < 0.001 |
| New AF | 1.15 | 1.09 – 1.21 | < 0.001 |
| Prior AF | 1.67 | 1.57 – 1.77 | < 0.001 |
| Males | 1.01 | 0.96 – 1.05 | 0.83 |
| Year of admission | 0.81 | 0.77 – 0.85 | < 0.001 |
| Referred from ED | 1.27 | 1.19 – 1.36 | < 0.001 |
| ICU admission | 1.01 | 0.96 – 1.08 | 0.64 |
| Hypertension | 0.98 | 0.94 – 1.02 | 0.32 |
| Hyperlipidaemia | 0.90 | 0.85 – 0.95 | < 0.001 |
| Diabetes | 1.36 | 1.30 -1.43 | < 0.001 |
| Current/ex-smoker | 1.12 | 1.08 – 1.17 | < 0.001 |
| Congestive heart failure | 1.50 | 1.42 – 1.57 | < 0.001 |
| Pulmonary embolism | 1.56 | 1.15- 2.10 | 0.004 |
| Deep venous thrombosis | 1.60 | 1.36 – 1.88 | < 0.001 |
| Ischaemic heart disease | 1.13 | 1.07 – 1.19 | < 0.001 |
| Prior CABG or PCI | 1.18 | 1.10 – 1.26 | < 0.001 |
| Ischaemic stroke | 1.70 | 1.51 – 1.91 | < 0.001 |
| Haemorrhagic stroke | 1.12 | 0.76 – 1.66 | 0.572 |
| Peripheral vascular disease | 1.28 | 1.20 – 1.36 | < 0.001 |
| Malignancy | 2.09 | 1.73 – 2.52 | < 0.001 |
| Sleep apnoea | 1.09 | 0.95 – 1.25 | 0.223 |
| Chronic kidney disease | 1.70 | 1.60 – 1.82 | < 0.001 |
| Chronic pulmonary disease | 1.52 | 1.42 – 1.63 | < 0.001 |
| Neurodegenerative disease | 1.64 | 1.35 – 1.99 | < 0.001 |
| Major bleed | 1.15 | 1.08 – 1.23 | < 0.001 |
| CABG | 1.08 | 1.03 – 1.14 | 0.002 |
| PCI | 1.57 | 1.22 – 2.02 | < 0.001 |
| Area of residence based on ASGSRA |  |  |  |
| Metropolitan | Reference |  | 0.648 |
| Inner regional | 0.99 | 0.94 – 1.04 | 0.637 |
| Outer regional / remote | 0.96 | 0.89 – 1.05 | 0.37 |
| IRSAD score | 0.999 | 0.999 – 0.999 | < 0.001 |
| AF status (No AF)*Type of cardiac valve surgery | Reference |  | 0.157 |
| AF status (New AF)*Type of cardiac valve surgery | 0.93 | 0.84 – 1.04 | 0.185 |
| AF status (Prior AF)*Type of cardiac valve surgery | 0.90 | 0.81 – 1.00 | 0.058 |
| AF, atrial fibrillation; aHR, adjusted hazard ratio; ASGSRA, Australian Statistical Geography Standard Remoteness Area; AVSx, aortic valve surgery; CABG, coronary artery bypass graft; CI, confidence interval; ED, Emergency Department; ICU, intensive care unit; IRSAD score, Index of Relative Socioeconomic Advantage and Disadvantage score; MACE, major adverse cardiovascular events defined as all-cause mortality or rehospitalisation with a primary diagnosis of acute myocardial infarction or ischemic cerebrovascular accident or congestive heart failure or revascularisation (percutaneous coronary intervention or coronary artery bypass graft), MVSx, mitral valve surgery; PCI, percutaneous coronary intervention. | | | |

**Supplementary Table S19. Multivariable Cox regression analysis of MACE in patients admitted for open-heart cardiac valve surgery who did not undergo CABG: whole study cohort**

| **Variables** | **aHR** | **95% CI** | **P value** |
| --- | --- | --- | --- |
| Age | 1.05 | 1.045 – 1.05 | < 0.001 |
| Study cohorts: AVSx vs MVSx | 0.86 | 0.81 – 0.91 | < 0.001 |
| AF status |  |  |  |
| No AF | Reference |  |  |
| New AF | 1.13 | 1.07 – 1.19 | < 0.001 |
| Prior AF | 1.71 | 1.61 – 1.82 | < 0.001 |
| Males | 0.96 | 0.91 – 1.01 | 0.093 |
| Year of Admission | 0.87 | 0.83 – 0.92 | < 0.001 |
| Referred from ED | 1.37 | 1.26 – 1.49 | < 0.001 |
| ICU admission | 1.01 | 0.94 – 1.08 | 0.83 |
| Hypertension | 1.05 | 1.00 – 1.11 | 0.039 |
| Hyperlipidaemia | 0.82 | 0.75 – 0.89 | < 0.001 |
| Diabetes | 1.35 | 1.27- 1.44 | < 0.001 |
| Current and ex-smoker | 1.10 | 1.05 – 1.15 | < 0.001 |
| Congestive heart failure | 1.50 | 1.40 – 1.60 | < 0.001 |
| Pulmonary embolism | 1.26 | 0.88 – 1.80 | 0.21 |
| Deep venous thrombosis | 1.55 | 1.25 – 1.92 | < 0.001 |
| Ischaemic heart disease | 1.16 | 1.09 – 1.23 | < 0.001 |
| Prior CABG or PCI | 1.51 | 1.39 – 1.64 | < 0.001 |
| Ischaemic stroke | 1.70 | 1.47 – 1.98 | < 0.001 |
| Haemorrhagic stroke | 1.31 | 0.86 – 2.01 | 0.21 |
| Peripheral vascular disease | 1.18 | 1.08 – 1.28 | < 0.001 |
| Malignancy | 1.71 | 1.33 – 2.21 | < 0.001 |
| Sleep apnoea | 1.23 | 1.05 – 1.44 | 0.01 |
| Chronic kidney disease | 1.64 | 1.50 – 1.78 | < 0.001 |
| Chronic pulmonary disease | 1.52 | 1.39 – 1.66 | < 0.001 |
| Neurodegenerative disease | 1.24 | 0.95 – 1.62 | 0.113 |
| Major bleed | 1.15 | 1.06 – 1.25 | 0.001 |
| PCI | 1.56 | 1.05 – 2.33 | 0.029 |
| Area of residence based on ASGSRA |  |  |  |
| Metropolitan | Reference |  | 0.463 |
| Inner regional | 0.97 | 0.91 – 1.03 | 0.27 |
| Outer regional / remote | 1.02 | 0.92 – 1.12 | 0.78 |
| IRSAD score | 0.99 | 0.999 – 0.999 | < 0.001 |
| AF, atrial fibrillation; aHR, adjusted hazard ratio; ASGSRA, Australian Statistical Geography Standard Remoteness Area; AVSx, aortic valve surgery; CABG, coronary artery bypass graft; CI, confidence interval; ED, Emergency Department; ICU, intensive care unit; IRSAD score, Index of Relative Socioeconomic Advantage and Disadvantage score; MACE, major adverse cardiovascular events defined as all-cause mortality or rehospitalisation with a primary diagnosis of acute myocardial infarction or ischemic cerebrovascular accident or cardiac failure or revascularisation (percutaneous coronary intervention or coronary artery bypass graft), MVSx, mitral valve surgery; PCI, percutaneous coronary intervention. | | | |

**Supplementary Table S20. Multivariable Cox regression analysis of MACE in patients admitted for open-heart cardiac valve surgery who did not undergo CABG: aortic valve surgery subgroup**

| **Variables** | **aHR** | **95% CI** | **P value** |
| --- | --- | --- | --- |
| Age | 1.05 | 1.04 – 1.05 | < 0.001 |
| AF status |  |  |  |
| No AF | Reference |  | < 0.001 |
| New AF | 1.17 | 1.10 – 1.25 | < 0.001 |
| Prior AF | 1.80 | 1.67 – 1.94 | < 0.001 |
| Males | 1.03 | 0.97 – 1.09 | 0.391 |
| Year of Admission | 0.84 | 0.79 – 0.90 | < 0.001 |
| Referred from ED | 1.34 | 1.21 – 1.49 | < 0.001 |
| ICU admission | 1.04 | 0.95 – 1.13 | 0.43 |
| Hypertension | 1.05 | 0.99 – 1.11 | 0.13 |
| Hyperlipidaemia | 0.81 | 0.73 – 0.89 | < 0.001 |
| Diabetes | 1.35 | 1.26 – 1.45 | < 0.001 |
| Current and ex-smoker | 1.08 | 1.02 – 1.15 | 0.010 |
| Congestive heart failure | 1.55 | 1.44 – 1.68 | < 0.001 |
| Pulmonary embolism | 1.42 | 0.85 – 2.37 | 0.18 |
| Deep venous thrombosis | 1.61 | 1.24 – 2.09 | < 0.001 |
| Ischaemic heart disease | 1.16 | 1.08 – 1.24 | < 0.001 |
| Prior CABG or PCI | 1.47 | 1.34 – 1.61 | < 0.001 |
| Ischaemic stroke | 1.46 | 1.20 – 1.77 | < 0.001 |
| Haemorrhagic stroke | 2.02 | 1.10 – 3.71 | 0.023 |
| Peripheral vascular disease | 1.11 | 1.01 – 1.22 | 0.040 |
| Malignancy | 1.82 | 1.33 – 2.48 | < 0.001 |
| Sleep apnoea | 1.34 | 1.11 – 1.61 | 0.002 |
| Chronic kidney disease | 1.61 | 1.46 – 1.78 | < 0.001 |
| Chronic pulmonary disease | 1.44 | 1.29 – 1.60 | < 0.001 |
| Neurodegenerative disease | 1.29 | 0.96 – 1.73 | 0.092 |
| Major bleed | 1.17 | 1.05 – 1.29 | 0.004 |
| PCI | 1.25 | 0.75 – 2.09 | 0.40 |
| Area of residence based on ASGSRA |  |  |  |
| Metropolitan | Reference |  | 0.81 |
| Inner regional | 0.99 | 0.92 – 1.06 | 0.73 |
| Outer regional / remote | 1.03 | 0.91 – 1.17 | 0.66 |
| IRSAD score | 0.999 | 0.999 – 0.999 | < 0.001 |
| AF, atrial fibrillation; aHR, adjusted hazard ratio; ASGSRA, Australian Statistical Geography Standard Remoteness Area; AVSx, aortic valve surgery; CABG, coronary artery bypass graft; CI, confidence interval; ED, Emergency Department; ICU, intensive care unit; IRSAD score, Index of Relative Socioeconomic Advantage and Disadvantage score; MACE, major adverse cardiovascular events defined as all-cause mortality or rehospitalisation with a primary diagnosis of acute myocardial infarction or ischemic cerebrovascular accident or cardiac failure or revascularisation (percutaneous coronary intervention or coronary artery bypass graft), MVSx, mitral valve surgery; PCI, percutaneous coronary intervention. | | | |

**Supplementary Table S21. Multivariable Cox regression analysis of MACE in patients admitted for open-heart cardiac valve surgery who did not undergo CABG: mitral valve surgery subgroup**

| **Variables** | **aHR** | **95% CI** | **P value** |
| --- | --- | --- | --- |
| Age | 1.05 | 1.045 – 1.054 | < 0.001 |
| AF status |  |  |  |
| No AF | Reference |  | < 0.001 |
| New AF | 0.994 | 0.89 – 1.11 | 0.92 |
| Prior AF | 1.49 | 1.34 – 1.66 | < 0.001 |
| Males | 0.85 | 0.78 – 0.92 | < 0.001 |
| Year of Admission | 0.91 | 0.82 – 1.01 | 0.077 |
| Referred from ED | 1.41 | 1.22 – 1.63 | < 0.001 |
| ICU admission | 0.95 | 0.85 – 1.07 | 0.42 |
| Hypertension | 1.07 | 0.98 – 1.17 | 0.15 |
| Hyperlipidaemia | 0.86 | 0.72 – 1.02 | 0.077 |
| Diabetes | 1.40 | 1.23 – 1.59 | < 0.001 |
| Current and ex-smoker | 1.12 | 1.03 – 1.22 | 0.011 |
| Congestive heart failure | 1.40 | 1.26 – 1.56 | < 0.001 |
| Pulmonary embolism | 1.19 | 0.71 – 1.97 | 0.51 |
| Deep venous thrombosis | 1.44 | 0.98 – 2.12 | 0.064 |
| Ischaemic heart disease | 1.17 | 1.04 – 1.31 | 0.009 |
| Prior CABG or PCI | 1.62 | 1.35 – 1.94 | < 0.001 |
| Ischaemic stroke | 2.12 | 1.67 – 2.68 | < 0.001 |
| Haemorrhagic stroke | 0.98 | 0.53 – 1.81 | 0.95 |
| Peripheral vascular disease | 1.50 | 1.25 – 1.81 | < 0.001 |
| Malignancy | 1.67 | 1.07 – 2.61 | 0.023 |
| Sleep apnoea | 0.96 | 0.70 – 1.31 | 0.80 |
| Chronic kidney disease | 1.67 | 1.41 – 1.96 | < 0.001 |
| Chronic pulmonary disease | 1.76 | 1.49 – 2.08 | < 0.001 |
| Neurodegenerative disease | 1.09 | 0.58 – 2.04 | 0.79 |
| Major bleed | 1.10 | 0.94 – 1.29 | 0.22 |
| PCI | 2.81 | 1.44 – 5.49 | 0.002 |
| Area of residence based on ASGSRA |  |  |  |
| Metropolitan | Reference |  | 0.32 |
| Inner regional | 0.92 | 0.83 – 1.03 | 0.14 |
| Outer regional / remote | 1.00 | 0.84 – 1.19 | 0.995 |
| IRSAD score | 0.99 | 0.998 – 0.999 | < 0.001 |
| AF, atrial fibrillation; aHR, adjusted hazard ratio; ASGSRA, Australian Statistical Geography Standard Remoteness Area; AVSx, aortic valve surgery; CABG, coronary artery bypass graft; CI, confidence interval; ED, Emergency Department; ICU, intensive care unit; IRSAD score, Index of Relative Socioeconomic Advantage and Disadvantage score; MACE, major adverse cardiovascular events defined as all-cause mortality or rehospitalisation with a primary diagnosis of acute myocardial infarction or ischemic cerebrovascular accident or cardiac failure or revascularisation (percutaneous coronary intervention or coronary artery bypass graft), MVSx, mitral valve surgery; PCI, percutaneous coronary intervention. | | | |

**Supplementary Table S22. Multivariable Cox regression analysis of all-cause mortality in patients admitted for open-heart cardiac valve surgery who did not undergo CABG: whole study cohort**

| **Variables** | **aHR** | **95% CI** | **P value** |
| --- | --- | --- | --- |
| Age | 1.07 | 1.06 – 1.07 | < 0.001 |
| Study cohorts: AVSx vs MVSx | 0.81 | 0.76 – 0.86 | < 0.001 |
| AF status |  |  |  |
| No AF | Reference |  | < 0.001 |
| New AF | 1.13 | 1.06 – 1.21 | < 0.001 |
| Prior AF | 1.60 | 1.50 – 1.72 | < 0.001 |
| Males | 1.02 | 0.97 – 1.08 | 0.48 |
| Year of Admission | 0.81 | 0.75 – 0.86 | < 0.001 |
| Referred from ED | 1.31 | 1.19 – 1.44 | < 0.001 |
| Hypertension | 0.99 | 0.93 – 1.04 | 0.62 |
| Hyperlipidaemia | 0.82 | 0.75 – 0.89 | < 0.001 |
| Diabetes | 1.35 | 1.26 – 1.45 | < 0.001 |
| Current and ex-smoker | 1.12 | 1.06 – 1.19 | < 0.001 |
| Congestive heart failure | 1.47 | 1.37 – 1.58 | < 0.001 |
| Pulmonary embolism | 1.36 | 0.90 – 2.04 | 0.143 |
| Deep venous thrombosis | 1.97 | 1.55 – 2.49 | < 0.001 |
| Ischaemic heart disease | 1.18 | 1.11 – 1.26 | < 0.001 |
| Prior CABG or PCI | 1.29 | 1.18 – 1.42 | < 0.001 |
| Ischaemic stroke | 1.83 | 1.56 – 2.16 | < 0.001 |
| Haemorrhagic stroke | 1.09 | 0.67 – 1.77 | 0.73 |
| Peripheral vascular disease | 1.20 | 1.09 – 1.32 | < 0.001 |
| Malignancy | 1.94 | 1.47 – 2.56 | < 0.001 |
| Sleep apnoea | 1.14 | 0.95 – 1.37 | 0.16 |
| Chronic kidney disease | 1.74 | 1.58 – 1.91 | < 0.001 |
| Chronic pulmonary disease | 1.56 | 1.41 – 1.72 | < 0.001 |
| Neurodegenerative disease | 1.58 | 1.20 – 2.09 | 0.001 |
| Major bleed | 1.17 | 1.07 – 1.29 | 0.001 |
| PCI | 1.57 | 1.02 – 2.43 | 0.042 |
| Area of residence based on ASGSRA |  |  |  |
| Metropolitan | Reference |  | 0.096 |
| Inner regional | 0.94 | 0.87 – 1.00 | 0.051 |
| Outer regional / remote | 0.92 | 0.82 – 1.04 | 0.179 |
| IRSAD score | 0.999 | 0.999 – 0.999 | < 0.001 |
| AF, atrial fibrillation; aHR, adjusted hazard ratio; ASGSRA, Australian Statistical Geography Standard Remoteness Area; AVSx, aortic valve surgery; CABG, coronary artery bypass graft; CI, confidence interval; ED, Emergency Department; ICU, intensive care unit; IRSAD score, Index of Relative Socioeconomic Advantage and Disadvantage score; MACE, major adverse cardiovascular events defined as all-cause mortality or rehospitalisation with a primary diagnosis of acute myocardial infarction or ischemic cerebrovascular accident or cardiac failure or revascularisation (percutaneous coronary intervention or coronary artery bypass graft), MVSx, mitral valve surgery; PCI, percutaneous coronary intervention. | | | |

**Supplementary Table S23. Multivariable Cox regression analysis of all-cause mortality in patients admitted for open-heart cardiac valve surgery who did not undergo CABG: aortic valve surgery subgroup**

| **Variables** | **aHR** | **95% CI** | **P value** |
| --- | --- | --- | --- |
| Age | 1.07 | 1.06 – 1.07 | < 0.001 |
| AF status |  |  |  |
| No AF | Reference |  | < 0.001 |
| New AF | 1.16 | 1.08 – 1.25 | < 0.001 |
| Prior AF | 1.67 | 1.53 – 1.81 | < 0.001 |
| Males | 1.08 | 1.01 – 1.16 | 0.02 |
| Year of Admission | 0.79 | 0.73 – 0.86 | < 0.001 |
| Referred from ED | 1.26 | 1.13 – 1.42 | < 0.001 |
| Hypertension | 0.98 | 0.92 – 1.05 | 0.55 |
| Hyperlipidaemia | 0.82 | 0.74 – 0.90 | < 0.001 |
| Diabetes | 1.37 | 1.26 – 1.48 | < 0.001 |
| Current and ex-smoker | 1.10 | 1.03 – 1.17 | 0.007 |
| Congestive heart failure | 1.49 | 1.36 – 1.62 | < 0.001 |
| Pulmonary embolism | 1.30 | 0.71 – 2.37 | 0.39 |
| Deep venous thrombosis | 2.12 | 1.60 – 2.80 | < 0.001 |
| Ischaemic heart disease | 1.19 | 1.10 – 1.29 | < 0.001 |
| Prior CABG or PCI | 1.23 | 1.11 – 1.36 | < 0.001 |
| Ischaemic stroke | 1.58 | 1.28 – 1.95 | < 0.001 |
| Haemorrhagic stroke | 1.21 | 0.61 – 2.39 | 0.59 |
| Peripheral vascular disease | 1.12 | 1.01 – 1.25 | 0.036 |
| Malignancy | 2.28 | 1.65 – 3.16 | < 0.001 |
| Sleep apnoea | 1.15 | 0.93 – 1.43 | 0.197 |
| Chronic kidney disease | 1.69 | 1.52 – 1.88 | < 0.001 |
| Chronic pulmonary disease | 1.44 | 1.28 – 1.61 | < 0.001 |
| Neurodegenerative disease | 1.63 | 1.19 – 2.22 | 0.002 |
| Major bleed | 1.17 | 1.05 – 1.31 | 0.007 |
| PCI | 1.34 | 0.78 – 2.29 | 0.29 |
| Area of residence based on ASGSRA |  |  |  |
| Metropolitan | Reference |  | 0.81 |
| Inner regional | 0.98 | 0.91 – 1.06 | 0.62 |
| Outer regional / remote | 0.96 | 0.84 – 1.11 | 0.60 |
| IRSAD score | 0.999 | 0.999 – 1.000 | 0.001 |
| AF, atrial fibrillation; aHR, adjusted hazard ratio; ASGSRA, Australian Statistical Geography Standard Remoteness Area; AVSx, aortic valve surgery; CABG, coronary artery bypass graft; CI, confidence interval; ED, Emergency Department; ICU, intensive care unit; IRSAD score, Index of Relative Socioeconomic Advantage and Disadvantage score; MACE, major adverse cardiovascular events defined as all-cause mortality or rehospitalisation with a primary diagnosis of acute myocardial infarction or ischemic cerebrovascular accident or cardiac failure or revascularisation (percutaneous coronary intervention or coronary artery bypass graft), MVSx, mitral valve surgery; PCI, percutaneous coronary intervention. | | | |

**Supplementary Table S24. Multivariable Cox regression analysis of all-cause mortality in patients admitted for open-heart cardiac valve surgery who did not undergo CABG: mitral valve surgery subgroup**

| **Variables** | **aHR** | **95% CI** | **P value** |
| --- | --- | --- | --- |
| Age | 1.07 | 1.06 – 1.08 | < 0.001 |
| AF status |  |  |  |
| No AF | Reference |  | < 0.001 |
| New AF | 1.01 | 0.89 – 1.16 | 0.84 |
| Prior AF | 1.44 | 1.26 – 1.64 | < 0.001 |
| Males | 0.90 | 0.82 – 1.00 | 0.044 |
| Year of Admission | 0.80 | 0.70 – 0.91 | < 0.001 |
| Referred from ED | 1.41 | 1.19 – 1.68 | < 0.001 |
| Hypertension | 0.99 | 0.89 – 1.11 | 0.87 |
| Hyperlipidaemia | 0.84 | 0.69 – 1.02 | 0.076 |
| Diabetes | 1.30 | 1.11 – 1.52 | 0.001 |
| Current and ex-smoker | 1.16 | 1.05 – 1.29 | 0.005 |
| Congestive heart failure | 1.44 | 1.27 – 1.63 | < 0.001 |
| Pulmonary embolism | 1.38 | 0.78 – 2.45 | 0.27 |
| Deep venous thrombosis | 1.62 | 1.05 – 2.49 | 0.028 |
| Ischaemic heart disease | 1.13 | 0.99 – 1.29 | 0.076 |
| Prior CABG or PCI | 1.53 | 1.24 – 1.87 | < 0.001 |
| Ischaemic stroke | 2.46 | 1.89 – 3.22 | < 0.001 |
| Haemorrhagic stroke | 0.94 | 0.46 – 1.90 | 0.86 |
| Peripheral vascular disease | 1.72 | 1.39 – 2.12 | < 0.001 |
| Malignancy | 1.48 | 0.87 – 2.51 | 0.15 |
| Sleep apnoea | 1.05 | 0.74 – 1.50 | 0.78 |
| Chronic kidney disease | 1.85 | 1.54 – 2.23 | < 0.001 |
| Chronic pulmonary disease | 1.99 | 1.65 – 2.39 | < 0.001 |
| Neurodegenerative disease | 1.46 | 0.78 – 2.73 | 0.24 |
| Major bleed | 1.20 | 1.00 – 1.43 | 0.045 |
| PCI | 2.86 | 1.34 – 6.12 | 0.007 |
| Area of residence based on ASGSRA |  |  |  |
| Metropolitan | Reference |  | 0.013 |
| Inner regional | 0.83 | 0.72 – 0.94 | 0.004 |
| Outer regional / remote | 0.88 | 0.71 – 1.09 | 0.24 |
| IRSAD score | 0.998 | 0.997 – 0.999 | < 0.001 |
| AF, atrial fibrillation; aHR, adjusted hazard ratio; ASGSRA, Australian Statistical Geography Standard Remoteness Area; AVSx, aortic valve surgery; CABG, coronary artery bypass graft; CI, confidence interval; ED, Emergency Department; ICU, intensive care unit; IRSAD score, Index of Relative Socioeconomic Advantage and Disadvantage score; MACE, major adverse cardiovascular events defined as all-cause mortality or rehospitalisation with a primary diagnosis of acute myocardial infarction or ischemic cerebrovascular accident or cardiac failure or revascularisation (percutaneous coronary intervention or coronary artery bypass graft), MVSx, mitral valve surgery; PCI, percutaneous coronary intervention. | | | |

**Supplementary Table S25. Risk of rehospitalisation for stroke stratified by type of open-heart cardiac valve surgery and atrial fibrillation status during admission:**

**Subgroup analyses according to CHA_2_DS_2_VASc group**

| **CHA_2_DS_2_VASc < 1** | | | |
| --- | --- | --- | --- |
| **Aortic valve surgery group** |  |  |  |
|  | **sHR** | **95% CI** | **P value** |
| New-AF vs No-AF reference group | 0.74 | 0.32 – 1.67 | 0.46 |
| Prior-AF vs No-AF reference group | 1.59 | 0.56 – 4.48 | 0.38 |
| **Mitral valve surgery group** |  |  |  |
|  | **sHR** | **95% CI** | **P value** |
| New-AF vs No-AF reference group | 1.24 | 0.58 – 2.65 | 0.59 |
| Prior-AF vs No-AF reference group | 1.65 | 0.71 – 3.84 | 0.25 |
| **CHA_2_DS_2_VASc ≥ 1** | | | |
| **Aortic valve surgery group** |  |  |  |
|  | **sHR** | **95% CI** | **P value** |
| New-AF vs No-AF reference group | 1.22 | 1.07 – 1.40 | 0.004 |
| Prior-AF vs No-AF reference group | 1.43 | 1.22 – 1.69 | <0.001 |
| **Mitral valve surgery group** |  |  |  |
|  | **sHR** | **95% CI** | **P value** |
| New-AF vs No-AF reference group | 0.93 | 0.75 – 1.15 | 0.52 |
| Prior-AF vs No-AF reference group | 1.11 | 0.90 – 1.38 | 0.32 |
| AF, atrial fibrillation; CI, confidence interval; sHR, subdistribution hazard ratio; SE, standard error.  Fine-Gray competing risk method was used to account for the competing risk of death. | | | |

**Supplementary Figure S1. Study cohort derivation**

All open cardiac valve surgical cases in CHeReL APDC database (n=40778 cases) (excluded 2,155 cases of non-NSW residents) (1-Jul-2001 - 31-Mar-2022)

n=35860 persons

n=19629 persons had isolated AVSx n=9916 persons had isolated MVSx

Final study cohort (1-Jul-2003 - 31-Mar-2021)

Isolated AVSx cohort (n=18949 persons)

No-AF (48%) ‡ (n=9017 persons)

New-AF (36%) ‡ (n=6874 persons)

Prior-AF (16%) ‡ (n=3058 persons)

Isolated MVSx cohort (n=9543 persons)

No-AF (35%) ‡ (n=3330 persons)

New-AF (36%) ‡ (n=3466 persons)

Prior-AF (29%) ‡ (n=2747 persons)

Limit to 1-Jul-2003 - 31-Mar-2021 for minimum 12 months follow-up to track outcomes (End-of-study follow-up: 31-Mar-2022)

Exclude prior prosthetic valve

Limit to ≥18yo

Limit to open-heart isolated AV or MV surgery only during admission †

Limit to index admission

Limit to 1-Jul-2003 - 31-Mar-2022 *

**Figure Legend**

Flow chart shows the derivation of the study cohort.

AF, atrial fibrillation; APDC, Admitted Patient Data Collection; AV, aortic valve; CHeReL, Centre for Health Record Linkage; NSW, New South Wales state of Australia; MV, mitral valve; Sx, surgery; yo, years old.

- This is performed to allow a “look-back” time-period of at least 24-months or more to assess for a prior history of AF
- Concomitant multivalve procedures (including on tricuspid or pulmonic valve) in single admission are excluded. Patients who had concomitant coronary artery bypass surgery is included.
- No-AF represents patients without AF; New-AF represents patients with new AF during index admission for cardiac valve surgery and no prior history of AF; Prior-AF represents patients with a history of AF.
